# Deletion of the murine ortholog of the *8q24* gene desert has anti-cancer effects in transgenic mammary cancer models

**DOI:** 10.1186/s12885-018-5109-8

**Published:** 2018-12-10

**Authors:** Collin Homer-Bouthiette, Yang Zhao, Lauren B. Shunkwiler, Benjamine Van Peel, Elizabeth Garrett-Mayer, Rachael C. Baird, Anna I. Rissman, Stephen T. Guest, Stephen P. Ethier, Manorama C. John, Patricia A. Powers, Jill D. Haag, Michael N. Gould, Bart M. G. Smits

**Affiliations:** 1 Department of Pathology and Laboratory Medicine, Medical University of South Carolina, 68 President Street, Charleston, SC 29425 USA; 2 Department of Public Health Sciences, Medical University of South Carolina, 135 Cannon Street, Charleston, SC 29425 USA; 3 Department of Oncology, McArdle Laboratory for Cancer Research, University of Wisconsin School of Medicine and Public Health, Madison, WI 53705 USA; 4 Department of Cell and Regenerative Biology, University of Wisconsin School of Medicine and Public Health, Madison, WI 53705 USA

**Keywords:** Breast cancer susceptibility, Noncoding, SNP, Risk, GWAS, Murine, PyMT, Mammary cancer - super-enhancer, Prevention

## Abstract

**Background:**

The gene desert on human chromosomal band *8q24* harbors multiple genetic variants associated with common cancers, including breast cancer. The locus, including the gene desert and its flanking genes, *MYC*, *PVT1* and *FAM84B*, is also frequently amplified in human breast cancer. We generated a megadeletion (MD) mouse model lacking 430-Kb of sequence orthologous to the breast cancer-associated region in the gene desert. The goals were to examine the effect of the deletion on mammary cancer development and on transcript level regulation of the candidate genes within the locus.

**Methods:**

The MD allele was engineered using the MICER system in embryonic stem cells and bred onto 3 well-characterized transgenic models for breast cancer, namely *MMTV-PyVT*, *MMTV-neu* and *C3(1)-TAg.* Mammary tumor growth, latency, multiplicity and metastasis were compared between homozygous MD and wild type mice carrying the transgenes. A reciprocal mammary gland transplantation assay was conducted to distinguish mammary cell-autonomous from non-mammary cell-autonomous anti-cancer effects. Gene expression analysis was done using quantitative real-time PCR. Chromatin interactions were evaluated by 3C. Gene-specific patient outcome data were analysed using the METABRIC and TCGA data sets through the cBioPortal website.

**Results:**

Mice homozygous for the MD allele are viable, fertile, lactate sufficiently to nourish their pups, but maintain a 10% lower body weight mainly due to decreased adiposity. The deletion interferes with mammary tumorigenesis in mouse models for luminal and basal breast cancer. In the *MMTV-PyVT* model the mammary cancer-reducing effects of the allele are mammary cell-autonomous. We found organ-specific effects on transcript level regulation, with *Myc* and *Fam84b* being downregulated in mammary gland, prostate and mammary tumor samples. Through analysis using the METABRIC and TCGA datasets, we provide evidence that *MYC* and *FAM84B* are frequently co-amplified in breast cancer, but in contrast with *MYC*, *FAM84B* is frequently overexpressed in the luminal subtype, whereas MYC activity affect basal breast cancer outcomes.

**Conclusion:**

Deletion of a breast cancer-associated non-protein coding region affects mammary cancer development in 3 transgenic mouse models. We propose *Myc* as a candidate susceptibility gene, regulated by the gene desert locus, and a potential role for *Fam84b* in modifying breast cancer development.

**Electronic supplementary material:**

The online version of this article (10.1186/s12885-018-5109-8) contains supplementary material, which is available to authorized users.

## Background

Mammalian genomes harbor large sequence regions (> 500-Kb) devoid of known protein-coding genes [1, 2]. Such sequences are widely known as ‘gene deserts’. Gene deserts encompass numerous gene regulatory elements, such as enhancers, and non-protein coding transcripts. Genome-Wide Association Studies (GWAS) over the last decade showed that gene deserts frequently harbor genetic variants associated with human complex traits, including breast cancer susceptibility. The gene desert located on human chromosomal band *8q24* is exemplary in that it contains multiple cancer-associated variants identified by GWAS, including 2 breast cancer-associated variants [3–5]. A recent fine-mapping study identified an additional independent association signal within the gene desert [6]. All 3 variants are common in the human population with risk allele frequencies of 0.41, 0.58, and 0.56, for rs13281615, rs1562430 (rs78152450), and rs35961416 respectively [3, 4, 6]. Variant rs13281615 is more strongly associated with the development of estrogen receptor (ER) positive (ER+) breast cancer as compared with ER negative (ER-) breast cancer [7, 8]. All polymorphisms strongly correlated to the tag Single Nucleotide Polymorphisms (SNPs) are located within non-protein coding sequences (the gene desert), suggesting their involvement in long-range gene regulation.

The genes located adjacent to the gene desert, *MYC* and *PVT1* at one side and *FAM84B* at the other side, are candidates to play a role in *8q24* variant-mediated breast cancer susceptibility. *MYC* is well-known as a proto-oncogene that encodes a transcription factor involved in many cellular processes such as cell growth, apoptosis, differentiation and protein translation [9]. *PVT1* is a long non-coding RNA (lncRNA) gene, but also produces at least 4 distinct microRNAs (miRNAs) [10]. The functions of these lncRNA transcripts and miRNAs are unknown. *FAM84B* (also known as *BCMP101*) has been implicated in breast cancer, as it was found in a proteomic analysis of cell membrane-associated proteins highly expressed in breast cancer [11]. The gene desert region also sires multiple lncRNAs, including *PNRCR1*, *CCAT1* and *POU5F1B* [12]. Although several of these lncRNAs have been shown to be involved in prostate or colorectal cancer susceptibility [13–15], none have been implicated in breast cancer thus far, reducing their candidacy potential. Chromosome conformation capture (3C)-based studies have identified higher-order chromatin structures connecting *MYC* and *PVT1* to the breast cancer-associated *8q24* gene desert region [16, 17]. Association of *MYC*, *PVT1*, or *FAM84B* transcript levels with any of the breast cancer risk alleles has not been reported. Global eQTL analysis on the The Cancer Genome Atlas (TCGA) data set for breast cancer did reveal numerous other transcripts associated with the rs418269 (in strong linkage disequilibrium with rs13281615) risk allele [18]. These transcripts were enriched with *MYC* binding motifs, suggesting that in breast tumors the risk allele (partially) acts through MYC regulation. In human primary colon, prostate and breast tumor tissue *MYC* transcript levels do not associate with the presence of the risk allele [19–22]. To show causal effects of this non-protein coding locus on gene expression and specific aspects of breast cancer etiology, a mammalian genetic model organism is essential.

**Fig. 1:**
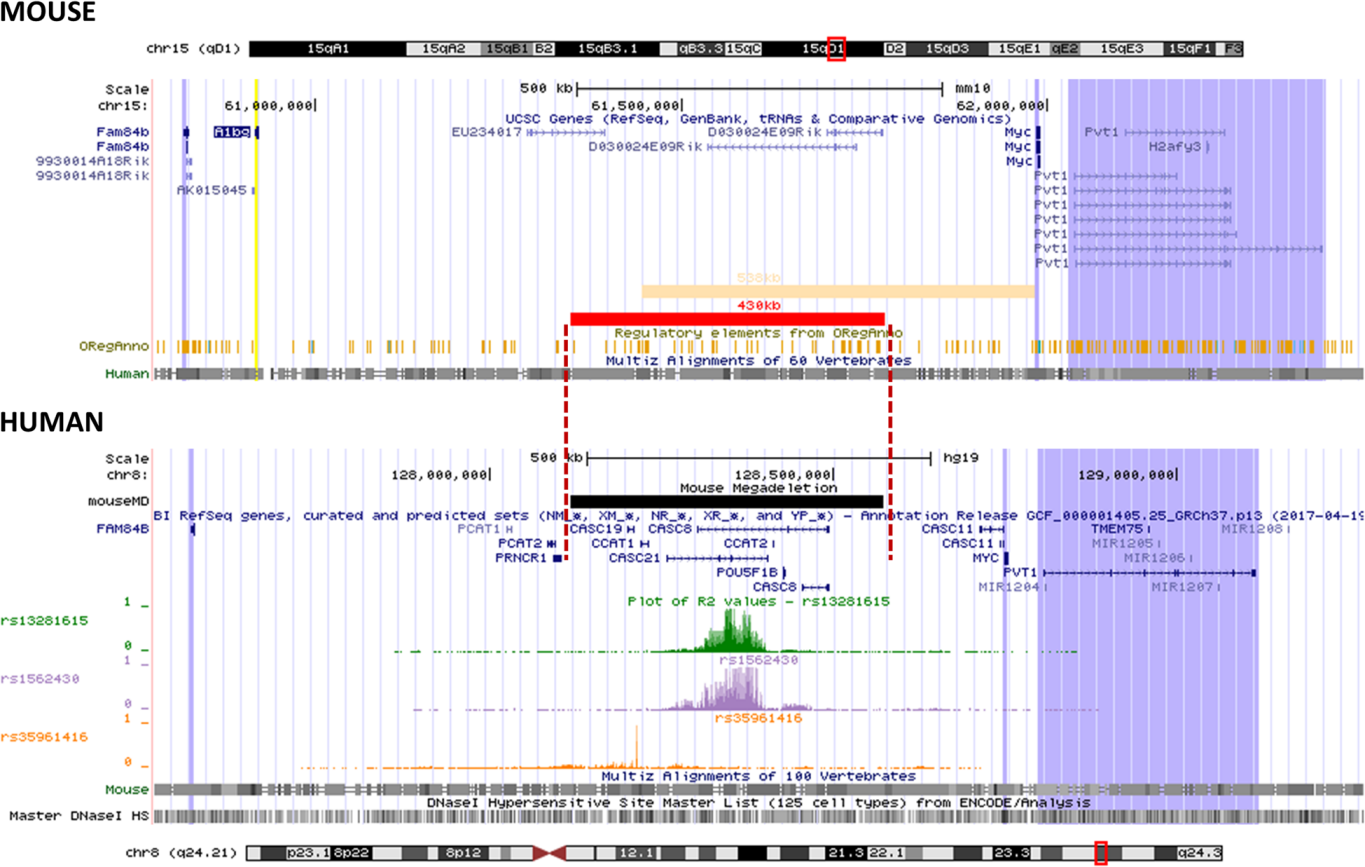
UCSC Genome Browser view of the mouse *15qD1* gene desert (upper) and orthologous human *8q24* cancer-associated gene desert (lower) proximal to the proto-oncogene, *MYC,* and distal to the gene, *FAM84b*. The mouse-human conserved protein-coding genes in the region are *Myc* and *Fam84b* indicated in purple shading. The conserved long non-coding RNA (lncRNA) gene, *Pvt1*, is also indicated in purple shading. The mouse *A1bg* gene (in light yellow shading) is not conserved in human and it is not expressed in mouse mammary gland. The human non-coding transcripts located in the gene desert (i.e. *PCAT1*, *PCAT2*, *PRNCR1*, *POU5F1B*, *CCAT1*, *CCAT2*, *CASC8*, *CASC11*, *CASC19*, *CASC21*) do not have mouse orthologs, although non-coding transcripts are produced from the mouse gene desert as well (i.e. *D030024E09Rik*, *Gm29904*, *Gm38563*, *4930402D18Rik*). The breast cancer-associated variants rs13281615, rs1562430, and rs35961416, and their respective correlated variants (level of r^2^ correlation in CEU population shown on y-axis) are indicated on separate tracks. The Multi-Z alignment tracks indicate good conservation from human to mouse, especially in the areas where the breast cancer variants with highest correlation level are located. The ORegAnno track (mouse) and DNAseI Hypersensitivity track (human) indicate potential gene regulatory elements. The red bar represents the mouse megadeletion (MD) interval (430-Kb). The deleted interval encompasses the region orthologous to the breast cancer-associated and correlated variants. The deletion interval recently generated in an unrelated study by Dave et al. is indicated in dark yellow [23]

The gene desert distal to *MYC* is considered orthologous between human and mouse, because (1) the flanking genes *MYC*, *PVT1* and *FAM84B* are conserved, (2) the total genomic range between *Myc* on one end of the gene desert and *FAM84B* on the other end is similar (1.2-Mb) in both species, (3) the non-coding region shows strong evolutionary sequence conservation between the 2 species, and (4) the presence of DNAseI Hypersensitivity (DNAseI HS) sites in the human ortholog and ORegAnno sites in the mouse ortholog is indicative of putative gene regulatory sequences in both species (Fig. 1).

Recently, Dave et al. published an analysis of a deletion mouse model lacking a 538-Kb region extending from the *Myc* gene promoter into the gene desert [23] (Fig. 1). This mouse model shows 50–80% downregulation of *Myc* expression and reduced development of several forms of cancer, including carcinogen-induced mammary tumorigenesis. While this study is of interest to link *Myc* downregulation to reduced cancer development, it does not specifically test the conserved breast cancer-associated region located in the middle of the gene desert proximal from *MYC*. We hypothesized that deletion of the conserved non-protein coding breast cancer-associated genomic region affects breast cancer development through regulation of candidate causal genes. We generated the MD allele resulting in deletion of approximately 430-Kb of sequence that encompasses all 3 regions orthologous to the breast cancer-associated variants in the *8q24* gene desert (Fig. 1). Our MD allele is different from the 538-Kb deletion described by Dave et al. in that it we deleted a gene desert region over 200-Kb proximal to the *Myc* gene promoter (Fig. 1).

The primary goal of the study was to test the effect of the deletion on mammary cancer development, by introducing the allele into well-characterized murine transgenic models for luminal, HER2+ and basal human breast cancer. The secondary goal was to identify candidate regulated genes, as the non-coding locus has been implicated in gene regulation. We determined the transcript levels of human-mouse conserved genes directly flanking the gene desert, namely *Myc*, *Pvt1* and *Fam84b*, as well as two other genes, *Trib1* and *Fam49b*, located on the same chromosomal band (*8q24* in human, *15qD1* in mouse) in RNA samples extracted from whole mammary gland, mammary tumors, prostate (all lobes combined), colon, bladder, spleen, and thymus tissue. These tissues were chosen, because variants associated with breast, prostate and colorectal cancer, as well as Chronic Lymphoblastic Leukemia (CLL), are located within the human ortholog of the deletion interval and one variant associated with urothelial/bladder cancer is located just outside of the deleted interval. The gene-specific expression studies highlighted *MYC* and *FAM84B* as strong candidate genes to be involved. *MYC* amplification has been previously implicated in breast cancer [24]. *FAM84B* amplification is a frequent event in esophageal squamous cell carcinoma [25]. How amplification and overexpression of the 2 genes relate to each other and to breast cancer outcomes is currently unknown. Analysis of publically available Molecular Taxonomy of Breast Cancer International Consortium (METABRIC) and TCGA data implies that *FAM84B* has effects on breast cancer that may be independent of *MYC*.

## Methods

### Mice

All mice are maintained in AAALAC-approved facilities at UW-Madison and MUSC. The protocol was approved by the Institutional Animal Care and Use Committee of the University of Wisconsin-Madison and of the Medical University of South Carolina. To minimize pain and distress, analgesics, anaesthesia and euthanasia were applied where indicated. None of the procedures described below resulted in unexpected death. Euthanasia was performed using carbon dioxide asphyxiation and cervical dislocation unless stated otherwise.

Generation of the MD mouse model was done using Mutagenic Insertion and Chromosome Engineering Resource (MICER) clone-assisted recombineering in embryonic stem (ES)-cells (AB2.2 from strain 129/SvEv) [26] as previously described [27]. Proper integration of the 2 clones was verified by Southern blot analysis (Additional file 1: Figure S1). Hypoxanthine/aminopterin/thymidine (HAT)-resistant, karyotypically normal ES-cell clones were monodispersed and microinjected into C57Bl/6 blastocysts to produce chimeric founders. After germ line establishment, the MD allele was introgressed onto the FVB/N genetic background for > 10 generations. All experiments were done on the FVB genetic background. MD carriers were intercrossed to generate homozygosity for the MD allele (MD−/−) or wildtype allele (MD+/+). Females and males were subjected to weight measurements. Organ weights were determined at necropsy (12 wks) and normalized to total bodyweight. Litter sizes were also recorded.

### Tumor phenotyping

Three well-characterized mouse models for human breast cancer were purchased (Jackson Labs), namely *MMTV-PyVT* (Polyoma Virus Middle-T antigen) [28], *MMTV-neu* (*HER2neu* oncogene) [29] and the *C3(1)-TAg* (C3(1)-Simian Virus 40 T antigen) [30], all existing on the FVB genetic background. MD−/− or MD+/+ mice were intercrossed with *MMTV-PyVT*, *MMTV-neu* or *C3(1)-TAg* to generate groups of carriers having no copies (*Transgene*;MD+/+), 1 copy (*Transgene*;MD+/−) or 2 copies (*Transgene*;MD−/−) of the MD allele. Latency to first palpable tumor, tumor growth (using digital caliper), overall survival, tumor multiplicity at necropsy and lung metastasis were recorded. For all models, the original FVB/NJ background and our FVB (containing 129/AJ) background (MD+/+), showed no difference in any tumor parameter measured (Additional file 1: Figure S2). Therefore, these subgroups were combined into one *MMTV-PyVT*;MD+/+, *MMTV-neu*;MD+/+ or *C3(1)-TAg*;MD+/+ group to compare against their respective MD−/− groups. Tumor-bearing mice were inspected twice weekly and measurements were recorded. In addition to inspection during tumor measurements, health monitoring was done at least once weekly by the veterinary care staff of the Division of Laboratory Animal Research at MUSC. Mice were euthanized when a tumor reached 2 cm in diameter or at humane endpoint when the mice show severe signs of distress, indicated by ulceration of a tumor, severe weight loss, abnormal reduced responsiveness to external stimuli, possibly in combination with hunched posture.

### Perfusion to assess lung metastasis

Tumor-bearing *MMTV-PyVT* and *MMTV-neu* mice were anesthetized using isoflurane and subjected to intracardiac perfusion with saline to drain the blood and blanch the lungs. Following euthanasia by thoractomy, a round gavage needle was inserted into the trachea to inflate the lungs with Amsterdam fixative. The lungs placed into 70% ethanol and visually inspected to quantify macroscopic metastatic foci present on all lobes. For histological analysis, lungs were removed from 70% ethanol, fixed in 4% paraformaldehyde for 48 h. Paraffin-embedded lung tissue was sectioned and stained with Hematoxylin and Eosin to visualize microscopic lung metastasis. Five sections taken at 50-μm intervals were quantified and averaged to calculate metastasis for each animal.

### Mammary gland transplantation assay

Under isoflurane anesthesia, the fat pads of MD+/+ and MD−/− recipients (21–28 days) were cleared by removing the growing mammary tree of both inguinal glands. Proper removal of host mammary tree was checked by whole mount analysis. Premalignant inguinal mammary glands from *MMTV-PyVT*;MD+/+ or *MMTV-PyVT*;MD−/− donors (28–35 days) were harvested, finely minced and grafted into both cleared recipient (MD+/+ or MD−/−) fat pads. Analgesics were given post-surgery for 2 days. Palpation of the graft sites began 4 weeks after surgery and occurred twice weekly. At humane end point, tumor end point or at 40 weeks after surgery, the transplant sites without tumors were whole mounted to examine graft rejection. Graft rejection was not detected in the *MMTV-PyVT*;MD+/+ donor groups, since whole mounts for transplant sites without a palpable tumor showed hyperplastic tissue and smaller tumor nodules. For the *MMTV-PyVT*;MD−/− donor groups, 3 out of 14 (MD+/+ recipient) and 2 out of 14 (MD−/− recipient) had at least 1 graft site rejected. Only mice without graft rejection were included in the analysis. Latency and lung metastasis were recorded as described above.

### Mammary gland whole mount analysis

From *MMTV-PyVT*;MD+/+ and *MMTV-PyVT*;MD−/− females at 4–5 weeks of age, and *C3(1)-TAg*;MD+/+ and *C3(1)-TAg*;MD−/− at 6 months of age, inguinal and thoracic mammary glands were removed, placed on slides and manipulated using blunt tweezers to thinly spread the fat pad. The whole mounted glands were then fixed and stained using standard Carmine Alum staining. Briefly, the slides were placed in 70% ethanol overnight, followed by an hour fixation in a solution containing 1 part glacial acetic acid and 3 parts 100% ethanol. Subsequently, the slides were washed in 70% ethanol, 50% ethanol and dH_2_O, for 15 min., 5 min. and 5 min., respectively. Then, the slides were stained in alum carmine solution (2.5 g alum potassium sulfate, 1.0 g carmine boiled in 500 ml dH_2_O) overnight, followed by washes in 70% ethanol, 95% ethanol and 100% ethanol, for 15 min. Each. Finally, the slides were de-stained in xylene until the mammary ductal structures are clearly visible. The stained whole mounts were photographed.

### Quantitative real-time PCR (QPCR) analysis of gene expression

Tissue samples were flash frozen in liquid nitrogen and stored at − 80 °C until further use. Frozen tissue samples were quickly homogenized in Tri-Reagent and total RNA was extracted using a total RNA extraction kit (Ambion) or chloroform-based extraction method. Following extraction, the RNA was DNAseI treated and visually inspected for DNA contamination and RNA degradation on an agarose gel. Total RNA (800 ng) was used as input in the reverse transcriptase reaction using the SSII system (Life technologies). QPCR was done using TaqMan assays (Life technologies), or using SYBR green assays with pre-tested primer sets (Integrated DNA Technologies; listed in Additional file 2: Table S1. TaqMan assay detection was done on a 9700 GeneAmp PCR system (Applied Biosystems) and SYBR detection was done on a Light Cycler 480 (Roche). The MD−/− and MD+/+ samples to be compared were always treated the same way and included in the same QPCR plate. Each measurement was done in triplicate. Only replicates within 1.0 Ct were retained and averaged to represent the sample value. Gene specific transcript levels were normalized by the 18S RNA level that served as an internal control.

### Chromosome conformation capture (3C) assay

Templates were prepared from isolated mammary epithelial cells from 6 MD+/+ mice, as previously described [31]. The restriction enzyme of choice was *Bgl*II. The fixed primer was chosen to be located in the *Myc* promoter. The experimental primers were chosen to be located within the deleted interval, biased towards regions of evolutionary sequence conservation. The relative interaction frequency for each experimental primer in combination with the fixed primer was determined for each sample as the average of at least 3 replicate measurements divided by the average of a Bacterial Artificial Chromosome (BAC)-based positive control template [31]. Primers and BACs are listed in Additional file 2: Table S1.

### METABRIC and TCGA analysis

The cBioPortal for Cancer Genomics was used to visualize and analyze the effect of *MYC* and *FAM84B* amplification and overexpression on breast cancer patient outcomes [32, 33]. Within the portal the METABRIC (2509 cases) or TCGA provisional (1105 cases) were selected. The data were queried using MYC and FAM84B as gene identifiers. Default settings were used to identify cases in which MYC or FAM84B were copy number amplified. To identify cases in which MYC or FAM84B were overexpressed, data were queried using an expression cutoff of greater than 2 standard deviations above the mean.

### Statistical analyses

Means of continuous variables were compared using two-sample t-tests or multifactorial ANOVA. Two-sided alpha level of 0.05 was used for determining statistical significance. Sample sizes are provided in figure captions. Survival curves on METABRIC and TCGA data were created using the built-in Kaplan-Meier Estimator and compared using the built-in log-rank tests.

## Results

### The megadeletion (MD) mouse model

The MICER clones for Cre-lox recombination in the ES cells were chosen to be located such that the deletion interval encompassed the mouse ortholog of the human region containing the breast cancer-associated and correlated variants (Fig. 1). The end of the resulting deletion interval is located approximately 200-Kb proximal to the *Myc* gene (Fig. 1). The human ortholog of the deleted sequence (dashed lines in Fig. 1, as determined by the Liftover function built in the UCSC Genome Browser) contains many DNAseI HS sites, indicative of putative gene regulatory sequences (Fig. 1), including those involved in the higher-order chromatin structures mentioned in the Introduction. In the mouse the deleted interval also contains many putative gene regulatory elements marked by ORegAnno track (Fig. 1).

**Fig. 2:**
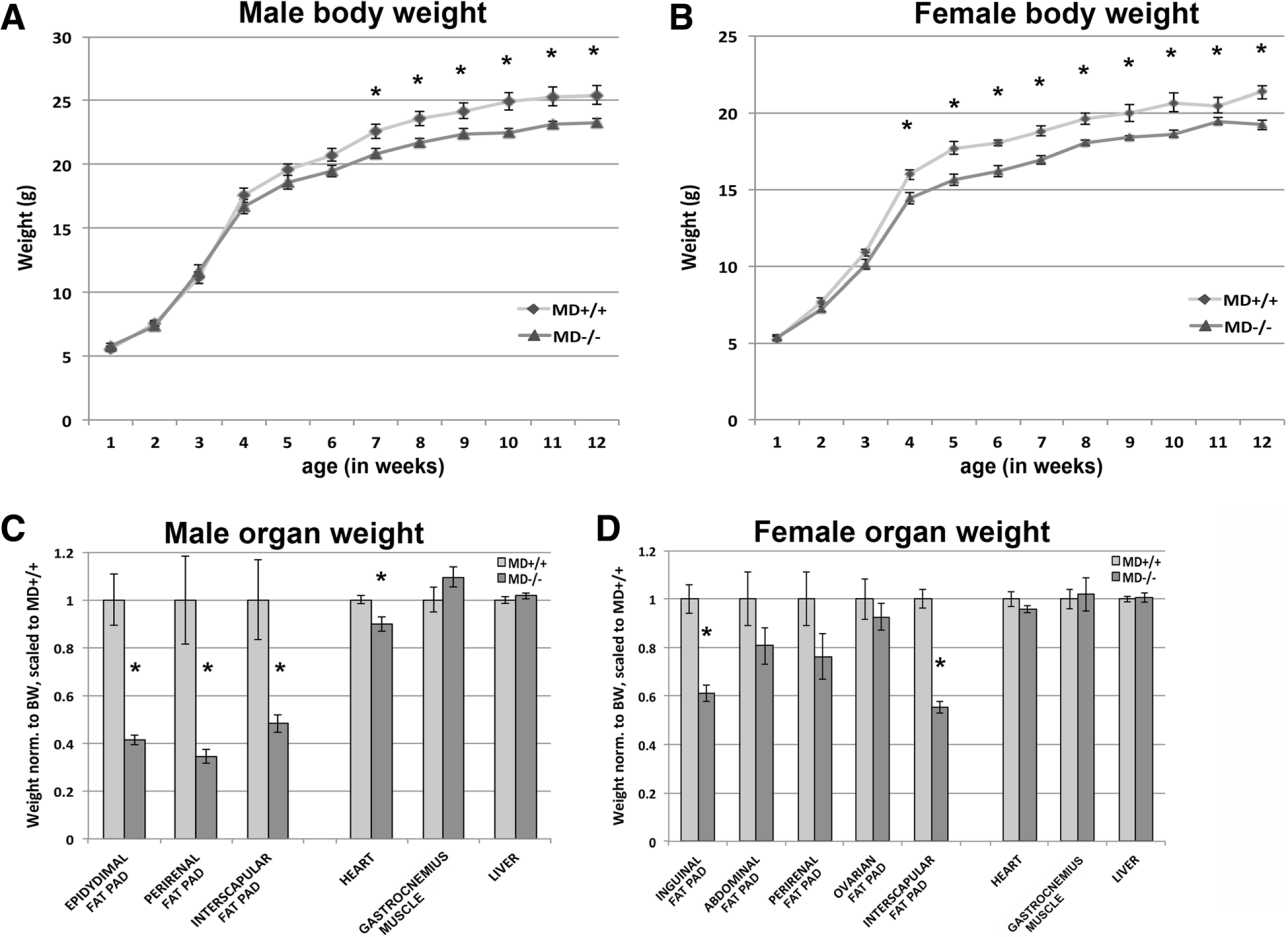
Weight measurements. **(a)** Male total bodyweight by age (MD+/+ *n* = 33, MD−/− *n* = 56). **(b)** Female total bodyweight by age (MD+/+ *n* = 44, MD−/− *n* = 48). **(c)** Male and **(d)** female organ weight measurements at 12 weeks of age (*n* = 6–14 each group for each organ), normalized to total bodyweight (BW). Epidydimal, perirenal and interscapular are the male fat pads measured. Inguinal (mammary), abdominal, perirenal, ovarian and interscapular indicate female fat pads. * = *p* < 0.05

Mice homozygous for the MD allele are viable. Litter sizes were not significantly different between breedings with MD−/− and MD+/+, indicating that viability and fertility were not affected (Additional file 1: Figure S1). The weights of female and male pups before weaning did not significantly differ between MD−/− and MD+/+ genotypes, indicating that lactation was not compromised. After weaning, the weights of both sexes of the MD−/− mice started to deviate from those of the MD+/+ mice resulting in a ~ 10% difference at 12 weeks (Fig. 2a, b). To trace the origin of this difference, we performed gross dissection of males and females at 12 weeks and weighed various fat pads and organs. The normalized (to total body weight) heart, liver and gastrocnemius muscle weight was not significantly different or showed no more than 10% difference between MD−/− and MD+/+ animals (Fig. 2c, d). In contrast, the normalized weights of the (inguinal) mammary and interscapular, fat pads were over 20% lower in MD−/− females than in MD+/+ females. The normalized weights of the abdominal, perirenal, and ovarian fat pads were lower, but did not reach statistical significance, suggesting potential compensatory mechanisms controlling for the sizes of those fat pads in the MD−/− female mice. In the males, the normalized weights of the epidydimal, perirenal and interscapular fat pads were over 50% lower in MD−/− than in MD+/+ mice (Fig. 2c, d). These data demonstrate that the deletion results in lower total body weight with adipose tissue disproportionally affected.

### Effects on mammary cancer development in transgenic mouse models for luminal, HER2+ and basal breast cancer

We crossed the allele onto 3 transgenic mouse breast cancer models, namely *MMTV-PyVT*, *MMTV-neu* and *C3(1)-TAg*. These models were chosen to represent 3 major subtypes of human breast cancer. The majority of *MMTV-PyVT*-induced tumors have been classified histologically and by their global gene expression profiles as luminal mammary tumors [34–36]. *MMTV-neu*-induced tumors represent mammary tumors of the HER2+ luminal subtype, and *C3(1)-TAg*-induced tumors represent tumors of the basal subtype of human breast cancer [34, 36]. The *MMTV-PyVT* and *MMTV-neu* models are also widely known as models for metastatic progression.

**Fig. 3:**
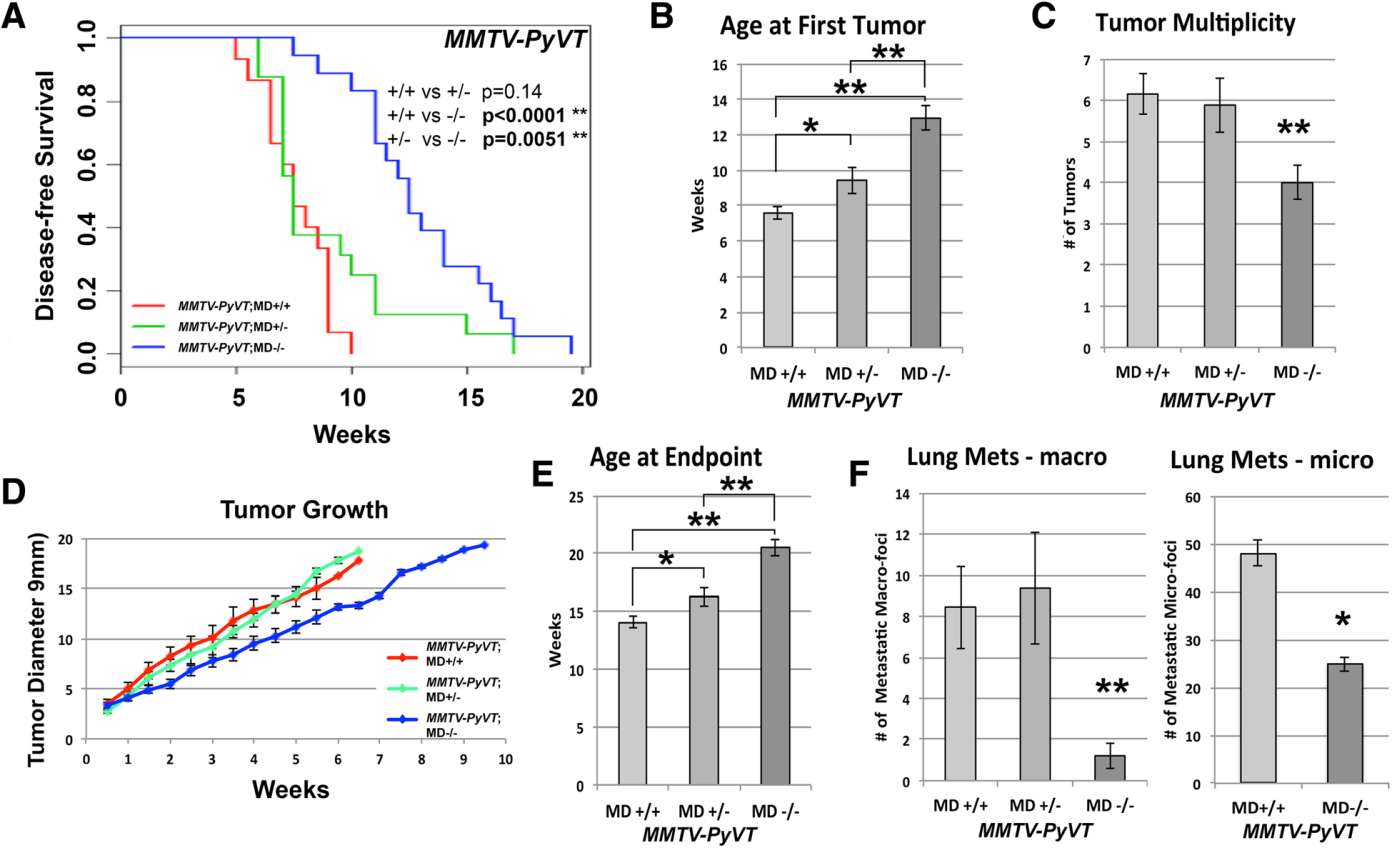
Modulation of tumorigenesis in the *MMTV-PyVT* transgenic model for human luminal breast cancer. **(a)** Disease-free survival curves for *MMTV-PyVT*;MD+/+ (*n* = 15), *MMTV-PyVT*;MD+/− (*n* = 16) and *MMTV-PyVT*;MD−/− (*n* = 18) mice. Log-rank test *P*-values are shown. **(b)** Average age at the appearance of the first palpable tumor. **(c)** Average tumor multiplicity at necropsy. **(d)** Tumor growth plot graphing average diameter size (in mm) versus weeks after first appearance. **(e)** Average age at end point. **(f)** Average amount of lung macro-metastatic foci (left panel). Average amount of lung micro-metastatic foci (right panel). * = p < 0.05, **p < 0.01

The mean latency to the first palpable tumor for *MMTV-PyVT*;MD+/+ was 7–8 weeks of age. *MMTV-PyVT*;MD+/− and *MMTV-PyVT*;MD−/− mice showed a significantly different disease-free survival curve (Fig. 3a). In *MMTV-PyVT*;MD−/− the mean latency was shifted to 13 weeks of age and in *MMTV-PyVT*;MD+/− to 9–10 weeks of age (Fig. 3b). *MMTV-PyVT*;MD−/− animals had a lower multiplicity at necropsy when compared to *MMTV-PyVT*;MD+/− and *MMTV-PyVT*;MD+/+ animals (Fig. 3c). The tumor growth curves reveal a tumor growth-reducing effect of *MMTV-PyVT*;MD−/−, but not *MMTV-PyVT*;MD+/−, as compared with *MMTV-PyVT*;MD+/+ (Fig. 3d). Increased latency and reduced tumor growth rate in the *MMTV-PyVT*;MD−/− animals resulted in increased overall survival (Fig. 3e). Despite the fact that age at necropsy was higher in the *MMTV-PyVT*;MD−/− animals than in the *MMTV-PyVT*;MD+/− and *MMTV-PyVT*;MD+/+ animals (allowing for more time to metastasize and grow), *MMTV-PyVT*;MD−/− animals have a ~ 4-fold reduced multiplicity of macro-metastatic foci than *MMTV-PyVT*;MD+/− and *MMTV-PyVT*;MD+/+ animals (Fig. 3f). Micro-metastatic foci multiplicity was also reduced (Fig. 3f), but less pronounced.

**Fig. 4:**
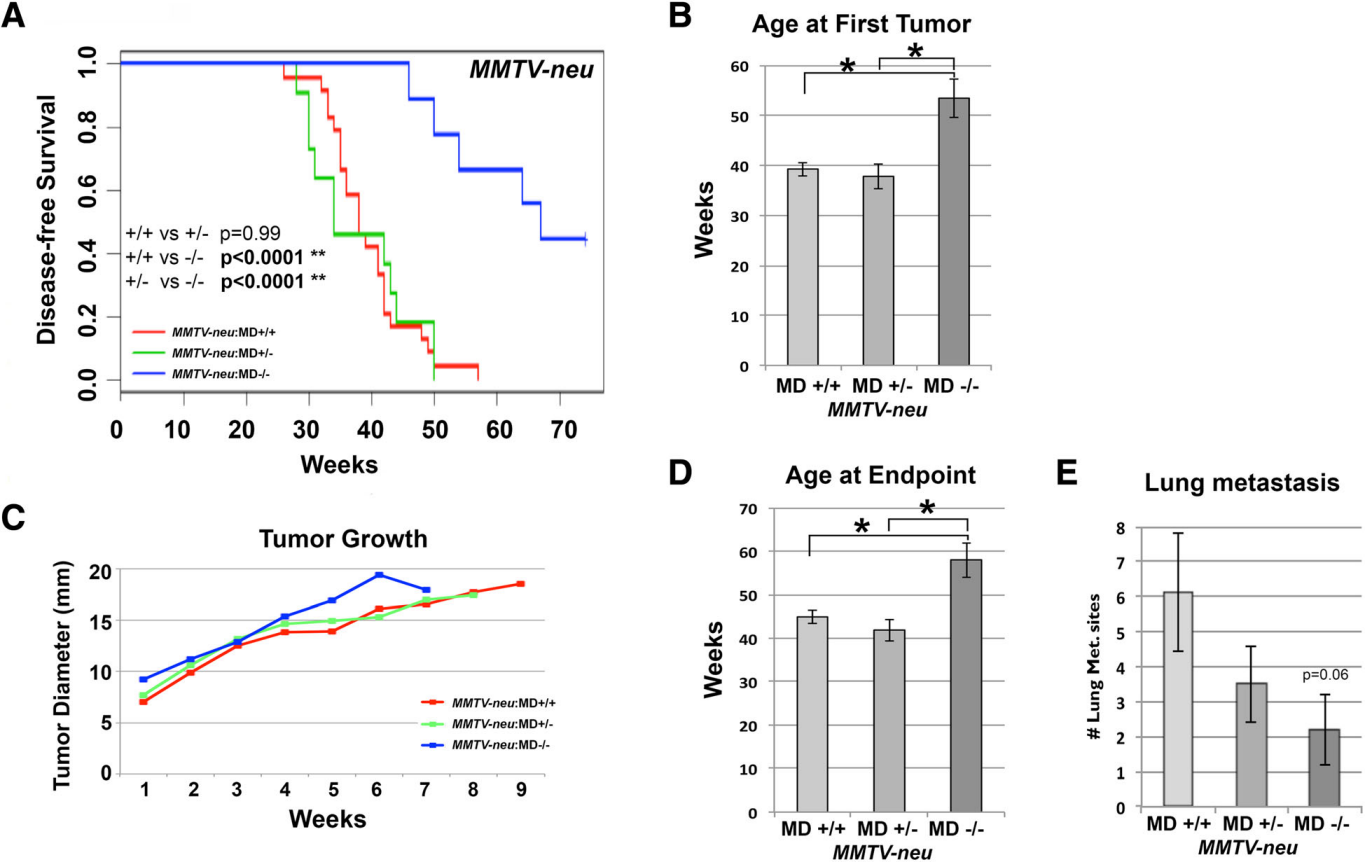
Modulation of tumorigenesis in the *MMTV-neu* transgenic model for human luminal HER2+ breast cancer. **(a)** Disease-free survival curves for *MMTV-neu*;MD+/+ (*n* = 19), *MMTV-neu*;MD+/− (*n* = 11) and MD−/− (n = 11) mice. Log-rank test P-values are shown. **(b)** Average age at the appearance of the first palpable tumor. **(c)** Tumor growth plot graphing average diameter size (in mm) versus weeks after first appearance. **(d)** Average age at end point. **(e)** Average amount of lung macro-metastatic foci. * = p < 0.05

The *MMTV-neu* carriers were palpated weekly starting at 16 weeks of age. Disease-free survival curves showed that *MMTV-neu*;MD−/− has a strong delay in tumorigenesis (Fig. 4a). Almost half of the mice were still tumor-free at 72 weeks of age. For the animals that did develop a tumor, latency was increased from 40 weeks of age for the *MMTV-neu*;MD+/+ group to 53 weeks of age for the *MMTV-neu*;MD−/− group (Fig. 4b). Since most mice developed only 1 tumor, multiplicity was not assessed. Tumor growth was not affected (Fig. 4c), which resulted in almost identical graphs for the average age at endpoint as for age at first tumor (Fig. 4d). In accordance with the *MMTV-PyVT* model, lung macro-metastatic foci multiplicity was also lower in *MMTV-neu*;MD−/− compared to *MMTV-neu*;MD+/+ (*p* = 0.06; Fig. 4e).

**Fig. 5:**
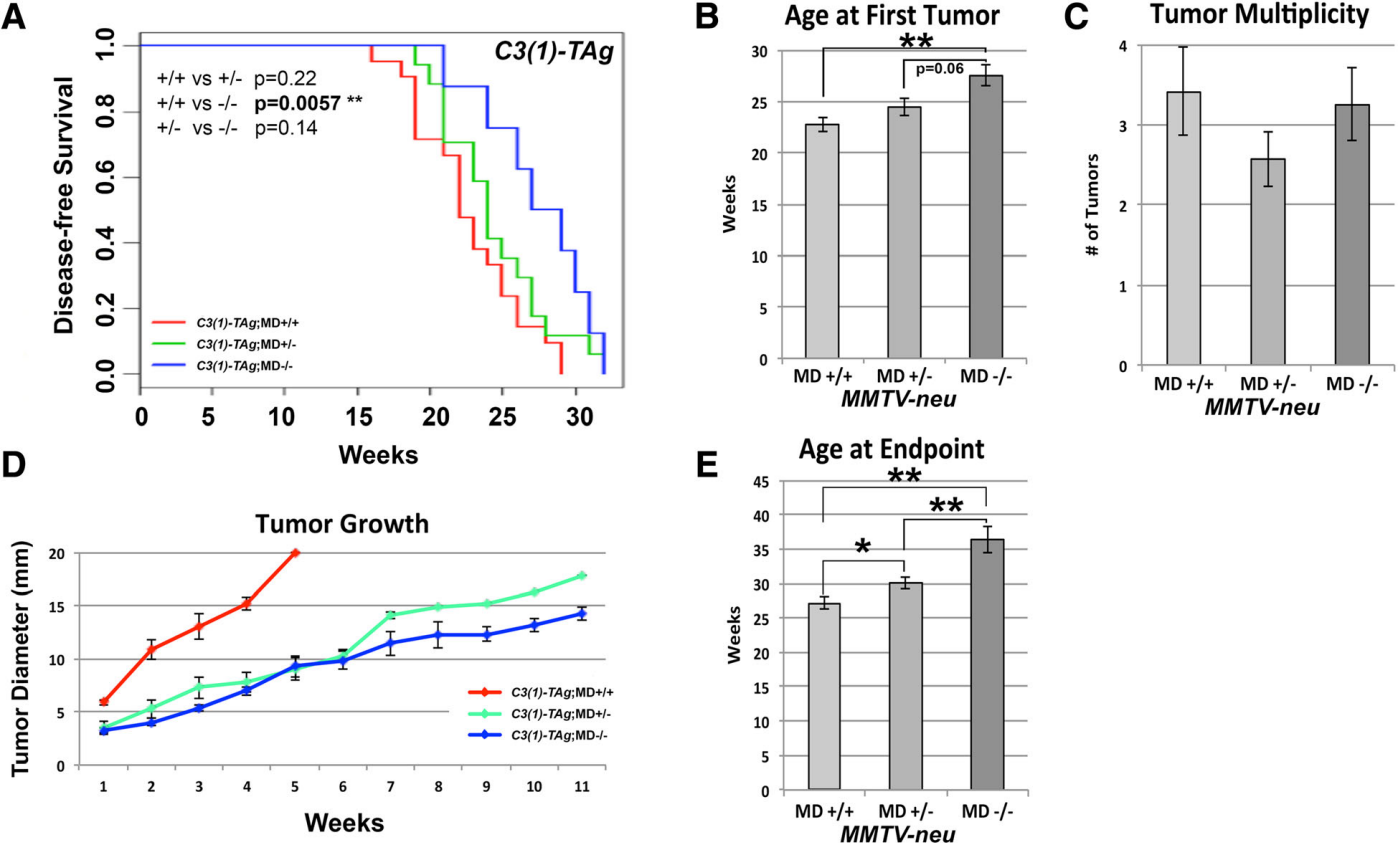
Modulation of mammary tumorigenesis in the *C3(1)-TAg* transgenic model for human basal breast cancer. **(a)** Disease-free survival curves for *C3(1)-TAg*;MD+/+ (*n* = 21), *C3(1)-TAg*;MD+/− (*n* = 17) and *C3(1)-TAg*;MD−/− (*n* = 8) mice. Log-rank test P-values are shown. **(b)** Average age at the appearance of the first palpable tumor. **(c)** Average tumor multiplicity at necropsy. **(d)** Tumor growth plot graphing average diameter size (in mm) versus weeks after first appearance. **(e)** Average age at end point. * = p < 0.05, **p < 0.01

The *C3(1)-TAg* carriers were palpated weekly starting at 12 weeks of age. Disease-free survival curves indicated that *C3(1)-TAg*;MD−/− mice show increased latency for *C3(1)-TAg*-induced tumorigenesis compared with *C3(1)-TAg*;MD+/+ mice (Fig. 5a). *C3(1)-TAg*;MD+/− mice were not different in disease-free survival from *C3(1)-TAg*;MD+/+ mice, but showed a strong non-significant trend (p = 0.06) to having decreased latency when compared with *C3(1)-TAg*;MD−/− (Fig. 5b). We did not observe an effect on tumor multiplicity in the *C3(1)-TAg* model (Fig. 5c). The tumor growth rate was affected by the MD allele in both heterozygous and homozygous state (Fig. 5d), which increased overall survival, as compared with *C3(1)-TAg*;MD+/+ (Fig. 5e). Metastasis incidence in this model is too low to be assessed.

We examined premalignant mammary glands by whole mount analysis in the *MMTV-PyVT* and *C3(1)-TAg* models at 4–6 weeks and 6 months of age, respectively. The protective effect of the MD allele on *MMTV-PyVT* -induced tumorigenesis is well visible at 46 days of age, since the *MMTV-PyVT*;MD+/+ glands show multifocal, dense hyperplasia, whereas the *MMTV-PyVT*;MD−/− glands show less advanced, more localized hyperplasia at that age (Additional file 1: Figure S3). A significantly lower amount of macroscopic hyperplastic nodules is observed in *MMTV-PyVT*;MD−/− mice as compared with *MMTV-PyVT*;MD+/+ mice (Additional file 1: Figure S3). These results indicate that the MD allele also affects early stages of tumor formation in the *MMTV-PyVT* model. In *C3(1)-TAg*, no significant difference in early lesions between MD+/+ and MD−/− at 6 months of age were found (Additional file 1: Figure S3), suggesting that the difference in latency in this model is mainly due to an effect on tumor progression.

Taken together, the MD allele intervenes with tumorigenesis at multiple stages, but in a transgene-specific manner, namely most prominently in the *MMTV-PyVT* mouse model for human luminal breast cancer and *MMTV-neu* mouse model for human HER2+ luminal breast cancer, but less prominently in the *C3(1)-TAg* mouse model for human basal breast cancer. This is in accordance with the human genetic epidemiological data where the *8q24* breast cancer-associated variant is strongly associated with ER+/luminal breast cancer and weaker with ER−/basal breast cancer [7].

### Mammary cell autonomy

**Fig. 6:**
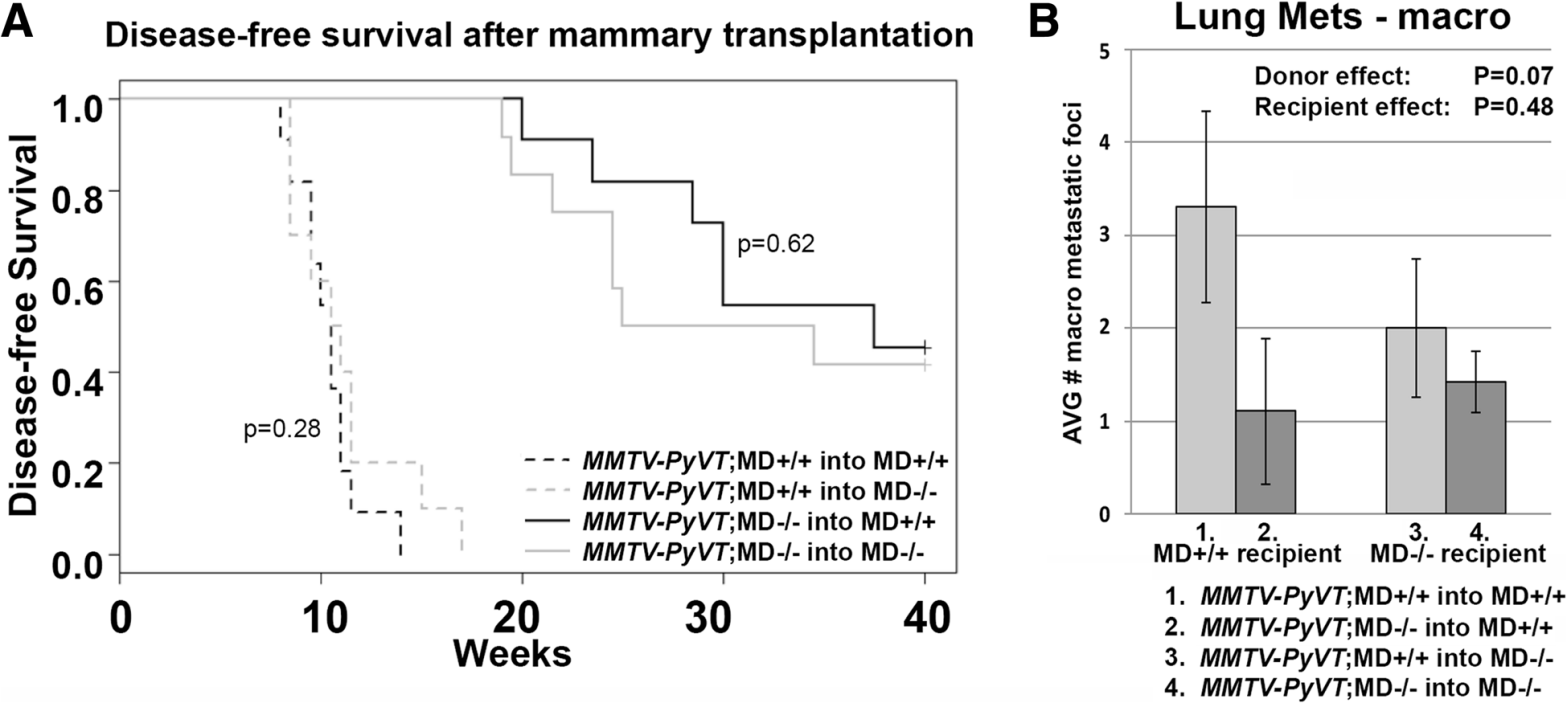
Reciprocal mammary gland transplantation assay. **(a)** Disease-free survival curves after transplantation for groups *MMTV-PyVT*;MD+/+ into MD+/+ (n = 11), *MMTV-PyVT*;MD+/+ into MD−/− (*n* = 10), *MMTV-PyVT*;MD−/− into MD+/+ (n = 11), and *MMTV-PyVT*;MD−/− into MD−/− (*n* = 12). P-values are for log-rank tests testing for the absence of a recipient (host) effect. **(b)** Average amount of lung macro metastatic foci per transplant group. P-values shown for 2-way ANOVA with dependent variables, donor and recipient

As we had observed a difference in mammary fat pad weight, we asked if the tumorigenesis phenotypes were autonomous to the mammary epithelium or if non-autonomous (host/micro-environment) factors were involved. Using a reciprocal transplantation assay, four transplant groups were generated, namely *MMTV-PyVT*;MD+/+ into MD+/+, *MMTV-PyVT*;MD+/+ into MD−/−, *MMTV-PyVT*;MD−/− into MD+/+ and *MMTV-PyVT*;MD−/− into MD−/−. Tumor-free survival curves showed a strong increase in latency between the *MMTV-PyVT*;MD+/+ and *MMTV-PyVT*;MD−/− donor groups (Fig. 6a). This finding indicates a strong mammary cell-autonomous (donor) effect of the MD allele on tumorigenesis. The log-rank test did not indicate a difference in the distribution of times to first tumor between the recipient genotypes, which means that the effect of the deletion on *MMTV-PyVT*-induced tumorigenesis is strictly mammary cell-autonomous.

While the effect of the MD allele on mammary tumor growth is autonomous, the effect on lung metastasis could show a recipient component. To determine this, lung macro-metastatic foci were quantified in recipients with palpable mammary tumors, small nodules and/or hyperplasia. Using two-way ANOVA analysis, we tested the null hypothesis that either donor and recipient genotypes or the interaction between donor and recipient have no effect on lung metastasis. We found that the donor x recipient interaction term was not significant (*P* = 0.28; Additional file 2: Table S2). The analysis suggests that there is a stronger donor effect (*P* = 0.07) than recipient effect (*P* = 0.49; Fig. 6b; Additional file 2: Table S2), suggesting that the difference in metastasis between *MMTV-PyVT*;MD+/+ and *MMTV-PyVT*;MD−/− mice likely has a mammary cell-autonomous component, but that a non-mammary cell-autonomous component cannot be completely ruled out.

### Expression regulation of mouse-human conserved candidate causal genes

**Fig. 7:**
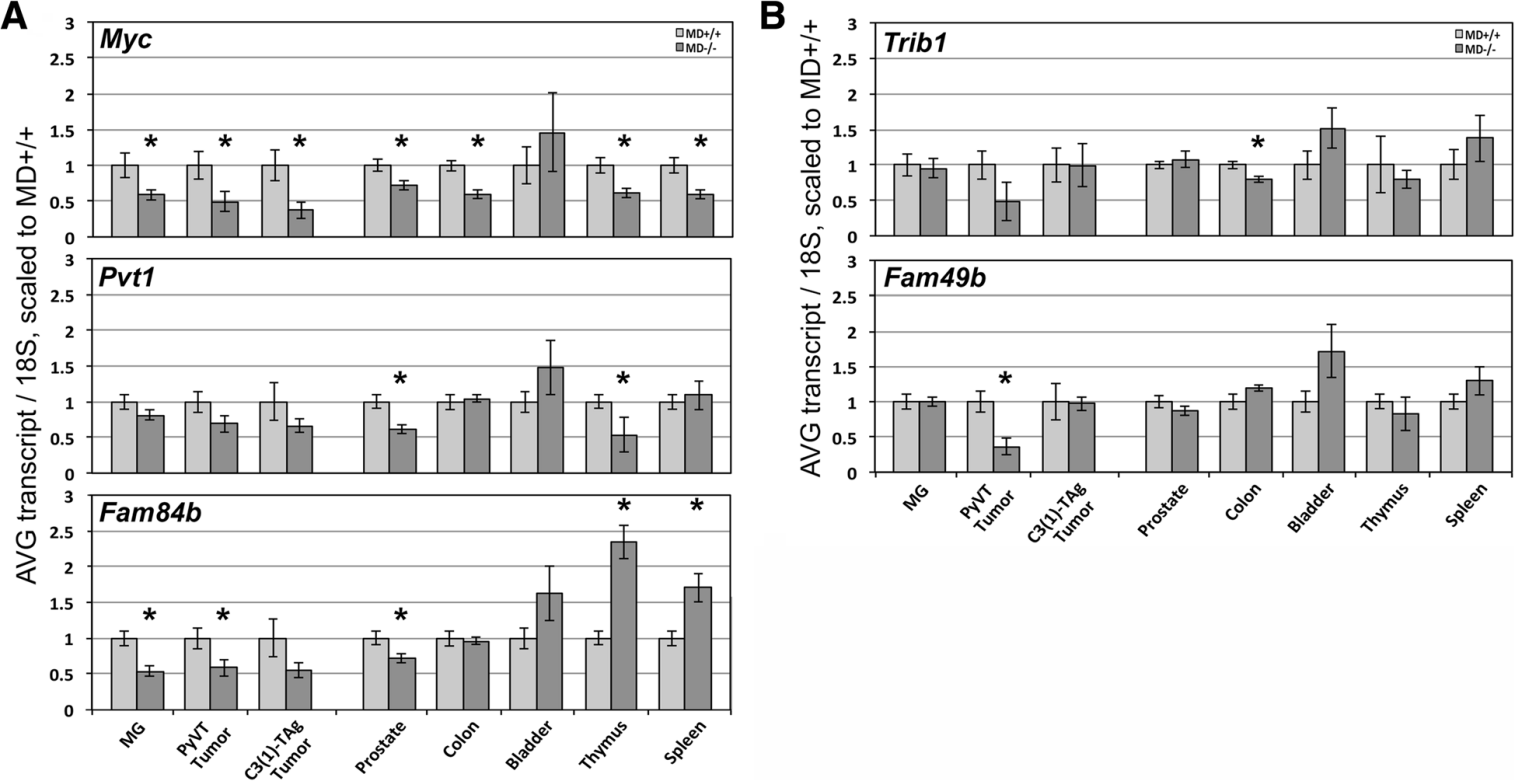
Gene expression analysis by quantitative real-time PCR (Q-PCR). **(a)** Transcript levels of *Myc*, *Pvt1* and *Fam84b*. Samples from mammary gland (MG, MD+/+ n = 19, MD−/− n = 21), *MMTV-PyVT*-induced mammary tumor (*MMTV-PyVT*;MD+/+ *n* = 9, *MMTV-PyVT*;MD−/− n = 12), *C3(1)-TAg*-induced mammary tumor (*C3(1)-TAg*;MD+/+ n = 9, *C3(1)-TAg*;MD−/− n = 6), prostate (MD+/+ n = 21, MD−/− n = 18), colon (MD+/+ *n* = 7, MD−/− n = 10), bladder (MD+/+ n = 7, MD−/− n = 10), thymus (MD+/+ *n* = 20, MD−/− n = 17) and spleen (MD+/+ *n* = 26, MD−/− *n* = 25) were used. **(b)** Transcript levels of *Trib1* and *Fam49b*. * = p < 0.05

We measured the transcript levels of mouse-human conserved candidate causal genes, *Myc*, *Fam84b*, *Pvt1*, *Trib1*, and *Fam49b*. The non-conserved non-coding mouse transcripts (*D030024E09Rik*, *EU234017*, *AK015045*, *9930014A18Rik*; Fig. 1) were not included in the analysis. Also not included was the non-conserved *A1bg* gene, as a poorly conserved human homolog is located on a different chromosome (chr19) in human and its expression cannot be detected in the mouse mammary gland. *Myc* transcript levels were reduced in MD−/− samples as compared with MD+/+ samples of all tissues examined, except the bladder (Fig. 7a). Most notably, *Myc* showed a reduction of approximately 40, 50 and 60% in the mammary gland, in *MMTV-PyVT*-induced and *C3(1)-TAg*-induced mammary tumors, respectively. *Pvt1* transcript levels showed a non-significant reduction in the mammary gland and mammary tumor samples. *Pvt1* in the prostate and thymus reached significance for a reduction of approximately 40%. In the colon, bladder and spleen, *Pvt1* was not differentially expressed. Similar to *Myc*, *Fam84b* showed a reduction of transcript level in the mammary gland and mammary tumor samples. *Fam84b* reduction was also observed in the prostate, but not in the colon or bladder. In contrast to the other genes, the transcript level of *Fam84b* was increased in the MD−/− versus MD+/+ samples in thymus and spleen. The transcript levels of *Trib1* and *Fam49b* were mostly equal between MD+/+ and MD−/−, except for a reduction of *Trib1* in the colon and a reduction of *Fam49b* in *MMTV-PyVT*-induced mammary tumors (Fig. 7b). The transcript levels of *Actb* were equal in all tissues measured (Additional file 1: Figure S4).

We checked if downregulation of transgene expression level could underlie the anti-cancer phenotypes. The *MMTV-PyVT*;MD−/− tumor samples showed an increased transgene level compared with *MMTV-PyVT*;MD+/+ tumor samples. In the *C3(1)-TAg* tumor samples the transgene expression level showed a non-significant increase in the MD−/− versus the MD+/+ samples (Additional file 1: Figure S5). These results suggest that the MD allele does not result in an anti-cancer effect through downregulation of the transgene itself.

Higher-order chromatin interactions connecting *MYC* to putative gene regulatory elements in the *8q24* gene desert have been identified in multiple studies [16, 37–39]. Using publically available global chromatin interaction data, generated through the Hi-C assay [40], we visualized the chromatin interaction heat map of the *8q24* gene desert locus along with the immediate neighboring genes for the human mammary epithelial cell line, HMEC (Additional file 1: Figure S5). The most prominent long-range interactions in the region involve *MYC*, including those previously found to bring the breast cancer-associated variants in proximity with *MYC* [39]. We then used the 3C assay to survey putative interactions between the *Myc* promoter and the MD interval in the mouse mammary gland. We found multiple sites within the interval interacting with *Myc* over a genomic distance of ~ 250–450-Kb. The *Myc*-promoter chromatin interaction profile for the human HMEC cell line and the mouse mammary gland appeared to be evolutionary conserved (Additional file 1: Figure S5).

### *MYC* and *FAM84B* gene copy number, expression, and association with clinical outcomes in 1105 primary breast tumors

**Fig. 8:**
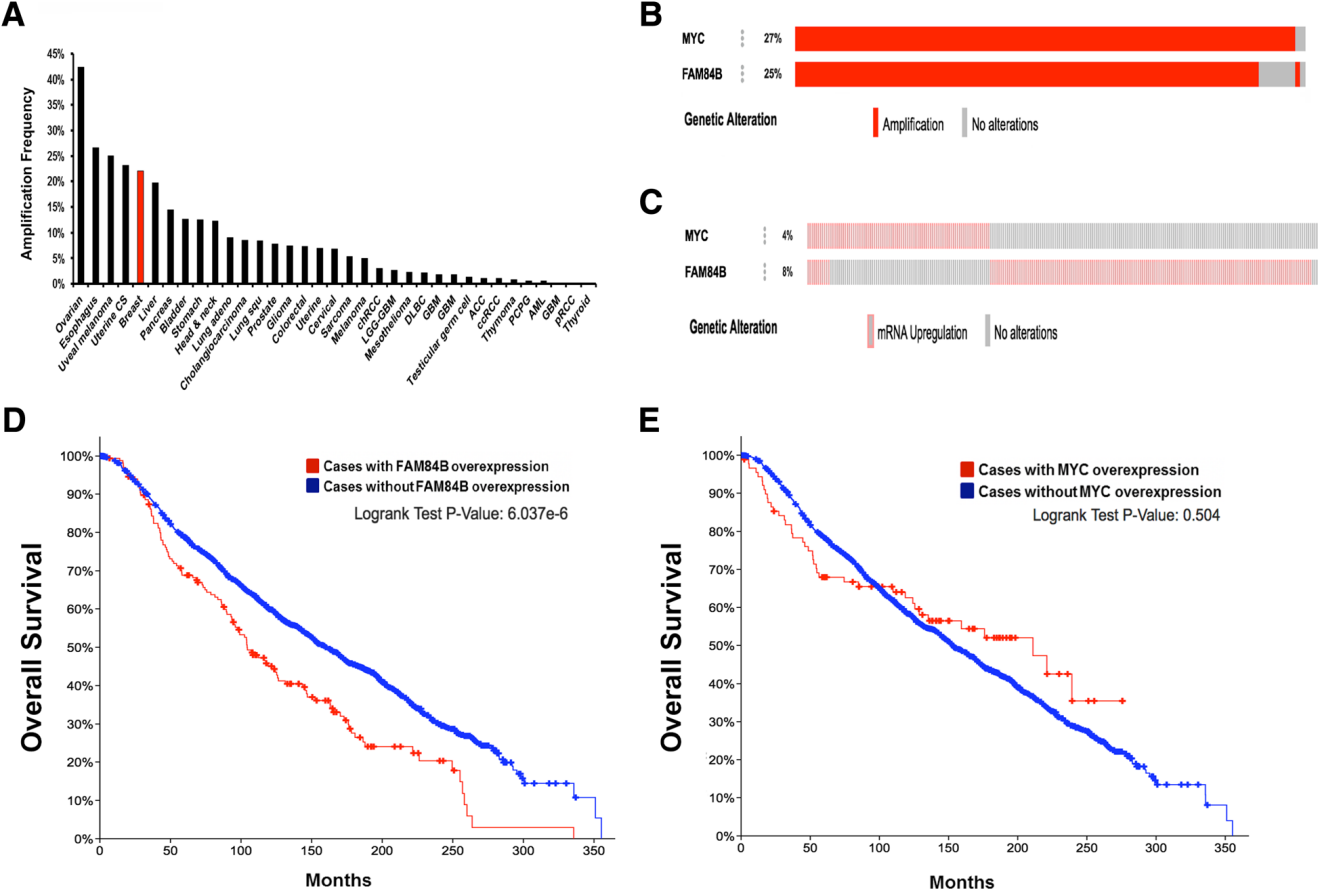
The association of *MYC* and *FAM84B* gene copy number increase and overexpression with clinical outcomes in 2509 primary breast cancers of the METABRIC data set. **(a)** Frequency of *MYC* and *FAM84B* copy number amplification in 35 cancer types from the TCGA bioportal database. **(b) (c)** Oncoprint showing cases from the METABRIC data with *MYC* or *FAM84B* gene amplification (**b**) or gene expression greater than 2 standard deviations (SD) above the mean (**c**). **(d) (e)** Overall survival plots from the cancer bioportal for METABRIC breast cancer cases with (red plot) or without (blue plot) *FAM84B* (**d**) or *MYC* (**e**) gene overexpression (> 2 SD)

We sought to investigate if mRNA expression levels of candidate genes *MYC* and *FAM84B* are associated with clinical outcomes in human breast cancer. The METABRIC and TCGA studies have generated comprehensive genomic portraits for invasive breast carcinomas [41, 42]. METABRIC is currently the most elaborate set with 2509 available cases, followed by TCGA that reported results for 1105 primary breast tumors. Querying the TCGA data through the cancer bioportal (http://www.cbioportal.org/) revealed that *MYC* and *FAM84B* are commonly amplified in multiple cancer types, including > 20% of all breast cancers (Fig. 8a). As METABRIC holds the most invasive breast cancer cases, we chose that data set for initial analysis. As expected, *MYC* and *FAM84B* are almost always co-amplified in the same breast tumors (Fig. 8b). However, examination of mRNA expression levels showed that *FAM84B* and *MYC* are only rarely overexpressed in the same breast tumor (Fig. 8c).

Examination of clinical outcomes showed that *FAM84B* overexpression was associated with a significant decrease in breast cancer patient overall survival (104.3 months vs 159.7 months; *P* = 6.037 × 10^− 6^; Fig. 8d), whereas *MYC* overexpression was not significantly correlated with decreased survival with decreased survival (210.9 months vs 152.3 months; *P* = 504; Fig. 8e). The TCGA data set shows a highly similar pattern, namely *FAM84B* overexpression was associated with a significant decrease in breast cancer patient overall survival (83.8 months vs. 129.6 months; *P* = 0.01; Additional file 1: Figure S6), whereas *MYC* overexpression was not significantly correlated (114.06 months vs. 122.8 months; *P* = 0.266; Additional file 1: Figure S6). This same pattern is also observed at the 1.5 SD cut-off in the TCGA data (Additional file 1: Figure S7). *FAM84B* is more commonly overexpressed than *MYC* in breast cancer, namely 9% of samples versus 4% of samples at 2 standard deviations (SD) above the mean for the METABRIC data (Fig. 8c) and 23% versus 7% of samples for the TCGA data set (Additional file 1: Figure S6). We checked if *MYC* might be more commonly overexpressed at a lower threshold (1.5 SD above the mean), but that was not found (Additional file 1: Figure S7). Interestingly, disease-free survival was not affected by *FAM84B* overexpression (Additional file 1: Figure S7). These results suggest that *FAM84B* may not affect latency to recurrence of the disease, but results in faster disease progression once recurrent disease is present.

## Discussion

The gene desert region proximal to *MYC* shows several key similarities between human and mouse, including similar organization of the genomic span, location of conserved genes (*Myc*, *Fam84b*, *Pvt1*), sequence conservation, and presence of putative gene regulatory elements. All non-coding transcripts and a coding gene (*A1bg*) located within the gene desert are not conserved between human and mouse. The *A1bg* gene is located distal to *Fam84b* in the mouse gene desert, but it aligns (by poor alignment scores) to a different human chromosome than the rest of the gene desert. In contrast to *Myc*, *Fam84b*, and *Pvt1*, *A1bg* is not detectable in mouse mammary gland samples and can therefore not be considered as a candidate.

In this study we characterized a novel mouse deletion allele that models the strong association of the human *8q24* locus with breast cancer susceptibility. The deletion spans the mouse ortholog of the human gene desert region associated with breast cancer risk. Even though 430-Kb of sequence was removed, the deletion does not result in reduced fertility, viability or lactation. The profound anti-tumorigenic effect of the deletion manifests at early and late stages of mammary cancer development in three transgenic mouse breast cancer models. The results indicate that in the *MMTV-PyVT* model the MD allele causes a reduction in mammary neoplastic nodules at 4–5 weeks of age, a strong delay in mammary tumor formation, a reduction in tumor progression and lower amount of metastatic foci in the lungs. In the *MMTV-neu* model the MD allele results in a strong delay in tumor formation, with some *MMTV-neu;*MD−/− not showing a tumor at all at 72 wks of age, as well as a reduction in metastatic foci in the lungs. In the *C3(1)-TAg* model a strong effect on tumor growth is observed, but the effect on latency is modest. These anti-cancer effects may be mouse model or transgene-specific, potentially due to an interaction of the deletion-regulated genes with transgene-specific oncogenic processes. The deletion is unlikely to act through downregulating expression of the transgene itself as in both the *MMTV-PyVT* and *C3(1)-TAg* models, the MD−/− samples showed higher transgene expression than the MD+/+ tumor samples. These observations suggest that higher transgene levels in the *transgene*;MD−/− tumors are needed to be transformative. Notably, the strongest effects on early tumorigenesis and latency are observed in the *MMTV-PyVT* and *MMTV-neu* models for luminal breast cancer progression and metastasis. This result is in accordance with the human epidemiological data showing a stronger association of one of the *8q24* variants with ER+/luminal breast cancer than with TNBC [7].

The transplantation assay indicated that despite an altered micro-environment (lower adiposity), the anti-cancer effects on tumor development elicited by the deletion allele are strictly intrinsic to the mammary cells. The transplantation assay was conducted essentially as described by Jackson et al. [43], but in contrast to our autonomous effect, their findings showed a non-autonomous effect of the genetic lesion on PyVT-induced mammary tumorigenesis, indicating that the assay can detect both autonomous and non-autonomous effects. We found the MD allele to be associated with a reduction of metastatic events in the *MMTV-PyVT* and *MMTV-neu* mouse models. The metastasis phenotype likely has a mammary cell-autonomous component in the *MMTV-PyVT* model, and may also have a non-mammary cell-autonomous component. There is ample evidence in the literature of micro-environment/host components affecting metastasis in the *MMTV-PyVT* model, most notably the involvement of T-cells, stroma, and macrophages [43–45].

We hypothesize that the non-coding deletion regulates gene expression in the mammary epithelium to control growth of the transformed cells arising in the mammary cancer-prone transgenic background. Our analysis revealed *Myc* and *Fam84b* as prominent candidate causal genes, as these were found to be differentially expressed between MD+/+ and MD−/− mammary gland and tumors samples, as well as various other tissue types. The proto-oncogene *MYC* encodes a transcription factor that together with its binding partner MAX is known to bind to E-box sequences to regulate gene expression involved in many cancer-related processes, including cell growth, proliferation, apoptosis, as well as general cellular processes, including transcription and translation [9]. Therefore, *MYC* is widely regarded as a target for regulation by the *8q24* variants. Chromatin looping from the *8q24* cancer risk-associated region to the *MYC* gene and *MYC* allelic imbalance have previously been implicated in the breast cancer-modulatory mechanism underlying the *8q24* locus [16–18, 39, 46]. However, *MYC* expression levels have not been found to be associated with any of the risk alleles. Our study shows that deletion of the murine ortholog of the human breast cancer-associated region within the gene desert locus, located approximately 200-Kb proximal to the *Myc* gene, results in a measurable reduction of *Myc* transcript level in the mammary gland and tumors. In accordance with this finding and the previously discussed genomic similarities and sequence conservation between the 2 species, we found mouse-human-conserved chromatin looping from the *Myc* promoter to the region orthologous to the human breast cancer-associated *8q24* region, suggesting that this gene desert region in both human and mouse contains regulatory elements affecting *Myc* expression in the mammary gland.

We found *Fam84b* to be downregulated to the same extent as *Myc*, suggesting that the deletion interval may contain regulatory elements for both *Myc* and *Fam84b*. *FAM84B* has also been identified to physically interact with the *8q24* gene desert locus through long-range chromatin looping, albeit in prostate cancer cells [47]. Because such higher-order chromatin structures have not been found in breast cells, we cannot conclude that *FAM84B* is a direct target for regulation by the deletion interval and could also be a secondary target resulting from *Myc* downregulation. *FAM84B* has been implicated in breast cancer development, since it was found in a screen to identify proteins associated with the cell membrane in breast cancer and was further shown to associate with adherens junctions [11]. The involvement of the lncRNA gene *Pvt1* in *8q24*-mediated breast cancer susceptibility has previously been suggested, although association of its expression level has been found to manifest with the risk allele of a breast cancer-associated SNP located within the *Pvt* locus [6], not within the *8q24* gene desert under study. We found a non-significant trend towards downregulation of *Pvt1* in the mammary glands and tumors of MD−/− mice, suggesting that the deletion interval may also contain *Pvt1* regulatory elements. Since, *Pvt1* downregulation was weaker than *Myc* and *Fam84b*, we hypothesize that *Pvt1* downregulation in the mammary gland is an indirect effect of *Myc* downregulation, as previously suggested [48].

Outside of the mammary gland and tumors, we show tissue-specific regulatory effects of the deletion on transcript levels of the genes surrounding the locus. Most notably, *Myc* transcript levels were reduced in the MD−/− samples in all tissues analyzed, except for bladder, suggesting the presence of *Myc*-enhancer elements within the deleted interval that act in tissues relevant to the human cancer associations with variants in the *8q24* gene desert.

Recently, Dave et al. published analysis of a 538-Kb deletion mouse model, lacking portion of our deletion interval, extending all the way to the *Myc* gene promoter [23]. Our MD allele is different because we deleted a genomic region over 200-Kb away from the *Myc* gene. Even though the deletions overlap only partially, our findings are mostly in accordance with the findings by Dave et al. Both studies find that a large deletion of the gene desert regulatory region is well tolerated by the organism, as no deleterious effects have been noticed. We found a ~ 10% reduction in total body weight in the homozygous MD mice, which was not reported by Dave et al. Strikingly, in both studies a strong, tissue-specific downregulation of *Myc* was observed along with anti-cancer properties against *Apc*^*min*^-induced polyp formation, carcinogen-induced mammary cancer [23], as well as transgene-induced luminal and basal mammary cancer (this study). No downregulation of *Myc* in the bladder was observed in both studies, and no effect on carcinogen-induced bladder cancer was found by Dave et al., suggesting that only in tissues with deletion-induced transcriptional silencing of *Myc*, tumorigenesis is disrupted. Given the striking overlap in phenotypes between the two deletion alleles, a genomic region controlling *Myc* regulation and anti-cancer properties can be assigned, namely the 333-Kb interval deleted in both mouse models (chr15:61445326–61,778,521 in mm10 built; Fig. 1). Dave et al. found that targeting individual conserved enhancer elements has limited effect on tumor development. Therefore, interrogating additional deletion mouse models genetically dissecting this gene desert further will result in a more detailed map of anti-cancer activity mediated by this locus.

*MYC* amplification has been known as a frequent genetic alteration in breast cancer for several decades [24]. Through the analysis of the TCGA data, it became clear that an increase in MYC activity and/or expression signature forms a subclass of TNBC (basal) breast cancer associated with poor outcome [49, 50]. We show here that, while the *8q24* amplicon is frequently observed in all breast cancers, an increased *MYC* transcript level is relatively uncommon and mostly occurring in the TNBC (basal) subtype. On the other hand, *FAM84B* transcript level increase occurs most frequently in the luminal and HER2+ subtypes and is associated with decreased overall survival in TCGA and METABRIC datasets, but not progression-free survival in TCGA (which is not available for METABRIC). These observations suggest that *FAM84B* has cancer-promoting properties contributing to poor outcome after recurrence, and that *MYC* is not the only oncogene located in this frequently amplified genomic region, supporting a role for the nearby *FAM84B* gene as a novel potential driver oncogene in human breast cancer. *Myc* and *Fam84b* may contribute synergistically and/or independently to the observed mammary cancer reduction phenotypes in the MD mouse model. This analysis will form the basis for additional mouse genetic model approaches in which more specific non-protein coding gene regulatory elements associated with breast cancer risk could be assessed for their effect on Myc and Fam84b regulation and breast cancer development. Other future work will be focused on deciphering the relationship between *Myc* and *Fam84b* overexpression in the development of specific breast cancer subtypes.

## Conclusions

Breast cancer risk variants identified by GWAS are mostly located to non-protein coding genomic regions. The gene desert on human chromosomal band *8q24* is a prototype example of a breast cancer-associated non-coding region. The cancer risk-associated interval contains multiple previously identified enhancer elements regulating nearby genes, most notably the proto-oncogene *MYC*. Upon deletion of the sequence in the mouse genome orthologous to the *8q24* gene desert region associated with breast cancer risk, we found that the deletion is well tolerated, as no deleterious effects were found in homozygous MD mice. The MD allele in homozygous state has anti-cancer effects in 3 transgenic mouse models for breast cancer. The results from a reciprocal transplantation assay suggest that the anti-cancer effects are initiated through activity of the deletion in cells of the mammary epithelium. We further show that deletion of the gene desert interval results in lower expression level of *Myc* and *Fam84b*, which are genes located adjacent to the gene desert, but over 200-Kb and 500-Kb away from the deletion interval, respectively. The expression study highlights these genes as strong candidates to be mediating the anti-cancer properties exhibited by the deletion. Our analysis of the METABRIC and TCGA datasets support the hypothesis that in human breast cancer, amplification and overexpression of *MYC* or *FAM84B* specifically contributes to development of the basal or luminal subtype, respectively.

## Additional files

Additional file 1: **Figure S1**: **A)** The Southern blots of the correctly inserted clones are shown in the panel. **B)** The MD allele in the mouse is viable in homozygous state and MD-/- mothers produce normal litter size, as compared with MD+/+ mothers. **Figure S2**: Comparison of tumor parameters for the PyVT **(A-D)**, C3(1)-TAg **(E-G)** and neu transgenes on the FVB/N genetic background (Jackson Labs) and our MD+/+ genetic background. No differences between the genetic backgrounds were detected. Hence, we added the data for the animals with the FVB/N genetic background to the MD+/+ groups for further analysis. **Figure S3**: Effect of MD allele on premalignant mammary glands in the PyVT and C3(1)-TAg models. **A**, **B)** Representative images of whole mounted mammary glands from PyVT MD+/+ (A) and PyVT MD-/- (B) mice at 46 days of age. The PyVT-induced hyperplastic expansion of the mammary epithelium is clearly visible in the MD+/+ glands, but strongly reduced in the MD-/- gland. **C**, **D)** Images of whole mounted mammary glands from C3(1)-TAg MD+/+ (A) and C3(1)-TAg MD-/- (B) mice at 5 months of age. Premalignant and malignant lesions are visible in both glands. **E**, **F)** Quantification of premalignant nodules and lesions on whole mounted mammary glands from PyVT MD+/+ (*n* = 9) and PyVT MD-/- (*n* = 4) mice (E), or C3(1)-TAg MD+/+ (*n* = 3) and C3(1)-TAg MD-/- (*n* = 4) mice (F), respectively. Graphed are averages +/- sem. **Figure S4**: Additional gene expression analysis. **A)** Comparison of housekeeping gene ActB transcript levels (normalized to 18S) between MD+/+ and MD-/- tissue samples. **B)** Comparison of PyVT or C3(1)-TAg transgene expression (normalized to 18S) between MD+/+ and MD-/- tumor tissue samples. Averages are shown, error bars are s.e.m. Significance (*p* < 0.05) is indicated by an asterisk. **Figure S5**: Higher-order chromatin interactions in the cancer-associated human and mouse gene desert. **A)** Higher-order chromatin interaction heat map of the 8q24 locus in human mammary epithelial cell line HMEC generated using the Jukebox tool. The positions of genes FAM84B, MYC and PVT1 are indicated. **B)** Chromatin interactions in the MD interval with the MYC promoter. The human interactions are shown in purple, the mouse in pink. The human interactions are derived from the vertical blue line in the heat map in panel A. The mouse interactions were determined experimentally, using the 3C assay on formaldehyde-fixed, restriction/ligation-treated MEC chromatin isolated from the mammary gland (MG) from MD+/+ mice. **Figure S6**: The association of MYC and FAM84B gene copy number increase and overexpression with clinical outcomes in 1105 primary breast cancers of the TCGA data set. **(A)** Frequency of MYC and FAM84B copy number amplification in 35 cancer types from the TCGA bioportal database. **(B**, **C)** Oncoprint showing cases from the TCGA database with MYC or FAM84B gene amplification (B) or gene expression greater than 2 standard deviations (SD) above the mean (C). **(D**, **E)** Overall survival plots from the TCGA bioportal for breast cancer patients with (red plot) or without (blue plot) FAM84B (D) or MYC (E) gene overexpression (> 2 SD). **Figure S7**: **A**, **B)** Overall survival plots from the TCGA cancer bioportal for breast cancer patients (*n* = 1105), with (red plot) or without (blue plot) FAM84B (A) or MYC (B) gene overexpression at > 1.5 SD. **C)** Disease-free survival plots from the TCGA bioportal for breast cancer patients with (red plot) or without (blue plot) FAM84B gene overexpression at > 2 SD. (DOCX 9763 kb)

Additional file 2: **Table S1.** List of primers, QPCR assays and BACs **Table S2.** 2-Way ANOVA for lung metastatic foci in PyVT mammary transplantation assay. **Table S3.** Total number of breast tumors in the TCGA database based on subtype. (DOCX 1631 kb)

## Abbreviations

3C: Chromosome Conformation Capture assay
AVG: Average
BAC: Bacterial Artificial Chromosome
CLL: Chronic Lymphoblastic Leukemia
ER: Estrogen Receptor
ES-cell: Embryonic stem-cells
EST: Expressed Sequence Tag
GWAS: Genome-Wide Association Study
HAT: Hypoxanthine/aminopterin/thymidine
Kb: Kilobase
LncRNA: Long non-coding RNA
MD: Megadeletion
METABRIC: Molecular Taxonomy of Breast Cancer International Consortium
MICER: Mutagenic Insertion and Chromosome Engineering Resource
miRNA: MicroRNA
PCR: Polymerase Chain Reaction
Q-PCR: Quantitative real-time PCR
SD: Standard deviation
Sem: Standard error of the mean
SNP: Single Nucleotide Polymorphism
TCGA: The Cancer Genome Atlas TNBC Triple Negative Breast Cancer
