# Supplementary material for: Deletion of the murine ortholog of the 8q24 gene desert has anti-cancer effects in transgenic mammary cancer models
Source: BMC Cancer. 2018 Dec 10;18:1233. doi: 10.1186/s12885-018-5109-8 (PMC6288875; doi:10.1186/s12885-018-5109-8)
Supplement: Supplementary file 1 — Figure S1: A) The Southern blots of the correctly inserted clones are shown in the panel. B) The MD allele in the mouse is viable in homozygous state and MD-/- mothers produce normal litter size, as compared with MD+/+ mothers. Figure S2: Comparison of tumor parameters for the PyVT (A-D), C3(1)-TAg (E-G) and neu transgenes on the FVB/N genetic background (Jackson Labs) and our MD+/+ genetic background. No differences between the genetic backgrounds were detected. Hence, we added the data for the animals with the FVB/N genetic background to the MD+/+ groups for further analysis. Figure S3: Effect of MD allele on premalignant mammary glands in the PyVT and C3(1)-TAg models. A, B) Representative images of whole mounted mammary glands from PyVT MD+/+ (A) and PyVT MD-/- (B) mice at 46 days of age. The PyVT-induced hyperplastic expansion of the mammary epithelium is clearly visible in the MD+/+ glands, but strongly reduced in the MD-/- gland. C, D) Images of whole mounted mammary glands from C3(1)-TAg MD+/+ (A) and C3(1)-TAg MD-/- (B) mice at 5 months of age. Premalignant and malignant lesions are visible in both glands. E, F) Quantification of premalignant nodules and lesions on whole mounted mammary glands from PyVT MD+/+ (n = 9) and PyVT MD-/- (n = 4) mice (E), or C3(1)-TAg MD+/+ (n = 3) and C3(1)-TAg MD-/- (n = 4) mice (F), respectively. Graphed are averages +/- sem. Figure S4: Additional gene expression analysis. A) Comparison of housekeeping gene ActB transcript levels (normalized to 18S) between MD+/+ and MD-/- tissue samples. B) Comparison of PyVT or C3(1)-TAg transgene expression (normalized to 18S) between MD+/+ and MD-/- tumor tissue samples. Averages are shown, error bars are s.e.m. Significance (p < 0.05) is indicated by an asterisk. Figure S5: Higher-order chromatin interactions in the cancer-associated human and mouse gene desert. A) Higher-order chromatin interaction heat map of the 8q24 locus in human mammary epithelial cell line HMEC generated using the [file 12885_2018_5109_MOESM1_ESM.docx]

**Additional file 1: FIGURES**

Collin Homer-Bouthiette^1,†^, Yang Zhao^1,†^, Lauren B. Shunkwiler^1^, Benjamine Van Peel^1^, Elizabeth Garrett-Mayer^2^, Rachael C. Baird^3^, Anna I. Rissman^1^, Stephen T. Guest^1^, Stephen P. Ethier^1^, Manorama C. John^4^, Patricia A. Powers^4^, Jill D. Haag^3^, Michael N. Gould^3^, Bart M. G. Smits^1,*^

^1^Department of Pathology and Laboratory Medicine, 68 President Street, Medical University of South Carolina, 29425 Charleston, SC, USA

^2^Department of Public Health Sciences, 135 Cannon Street, Medical University of South Carolina, 29425 Charleston, SC, USA

^3^McArdle Laboratory for Cancer Research, Department of Oncology, University of Wisconsin School of Medicine and Public Health, 53705 Madison, WI, USA

^4^Department of Cell and Regenerative Biology, University of Wisconsin School of Medicine and Public Health, 53705 Madison, WI, USA

*To whom correspondence should be addressed. Tel: +1 843 876 2293; E-mail: [smitsb@musc.edu](mailto:smitsb@musc.edu)

^†^ These authors contributed equally to this work

**INTRODUCTION TO SUPPLEMENTARY FIGURES**

**MICER clone genetic engineering:** The 430-Kb megadeletion was accomplished using Mutagenic Insertion and Chromosome Engineering Resource (MICER) clone-assisted recombineering in mouse embryonic stem (ES)-cells. To this end, *loxP*-sites were inserted using consecutive MICER-clone-based targeting at predefined sites on the same chromosome. One MICER clone (MHPP-284c18; 3’HPRT) was purchased (Sanger Institute, UK) and one MICER clone was generated by cloning a genomic insert into the *Asc*I restriction site of the MPHN (5’HPRT) backbone vector (Primer sequences in Additional File 2: Table S1). The *loxP*-sites flank the cancer-associated area in the murine *8q24* gene desert ortholog. The interval was excised using Cre-recombinase transfection of correctly targeted ES-cells, creating the Megadeletion (MD) allele. The allele was generated in 129/Av ES-cells, microinjected into C57BL/6 blastocysts and backcrossed to the FVB/N genetic background for 10+ generations. A homozygous MD (MD-/-) line and a wild type control (MD+/+) line were derived following backcrossing, providing groups of animals with similar genetic background for experiments. Average litter sizes of breedings with the MD-/- and MD+/+ lines are plotted in the panel.

**Higher-order chromatin interactions:** The Juicebox tool was used to visualize ligation-based proximity mapping data generated by Hi-C. Due to random ligations chromatin interactions are frequent between neighboring genomic fragments, which creates the dark red diagonal in the heat map. A local hot spot at further genomic distance (away from the diagonal) indicates long-range interactions potentially informing gene regulatory events. The grey area represents the human ortholog of the mouse megadeletion (MD) interval. The pink area represents the haplotype blocks associated with human breast cancer. The interaction pattern suggests that in this cell line *FAM84B* is located in a different chromosomal domain (or globule) than the MD interval, *MYC* and *PVT1*. In addition, the *MYC* gene interacts frequently with fragments within the gene desert (indicated with a vertical blue line), including fragments within the breast cancer-associated haplotype, suggesting the presence of long-range gene regulatory elements.

To detect ligation events, indicative of chromatin interactions, a fixed primer in the *MYC* promoter was used together with primers along the MD interval. The PCR band intensity is normalized to the PCR band intensity obtained from a randomly ligated (BAC-based) control sample, resulting in a relative interaction frequency profile. The average of 6 independent templates is shown. The plot shows that higher-order chromatin interactions between the *MYC* promoter and the MD interval are concentrated within the breast cancer-associated haplotype (in pink shading) in human and mouse MECs. The chromatin interaction data suggest that the approximate locations of the long-range *Myc* regulatory elements involving the breast cancer-associated region are conserved between mouse and human mammary cells.


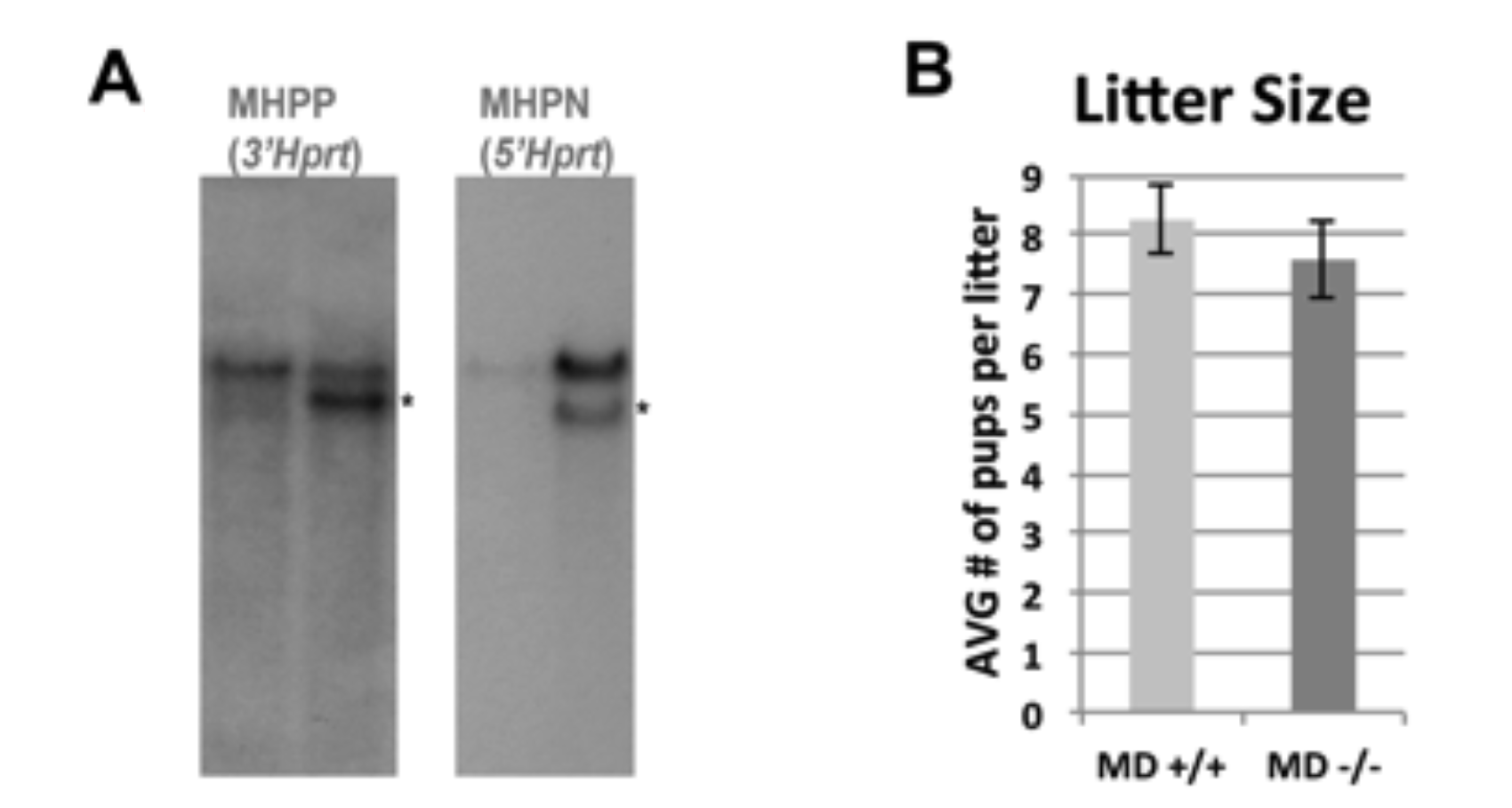


**Additional file 1: Figure S1:** **A)** The Southern blots of the correctly inserted clones are shown in the panel. **B)** The MD allele in the mouse is viable in homozygous state and MD-/- mothers produce normal litter size, as compared with MD+/+ mothers.

**
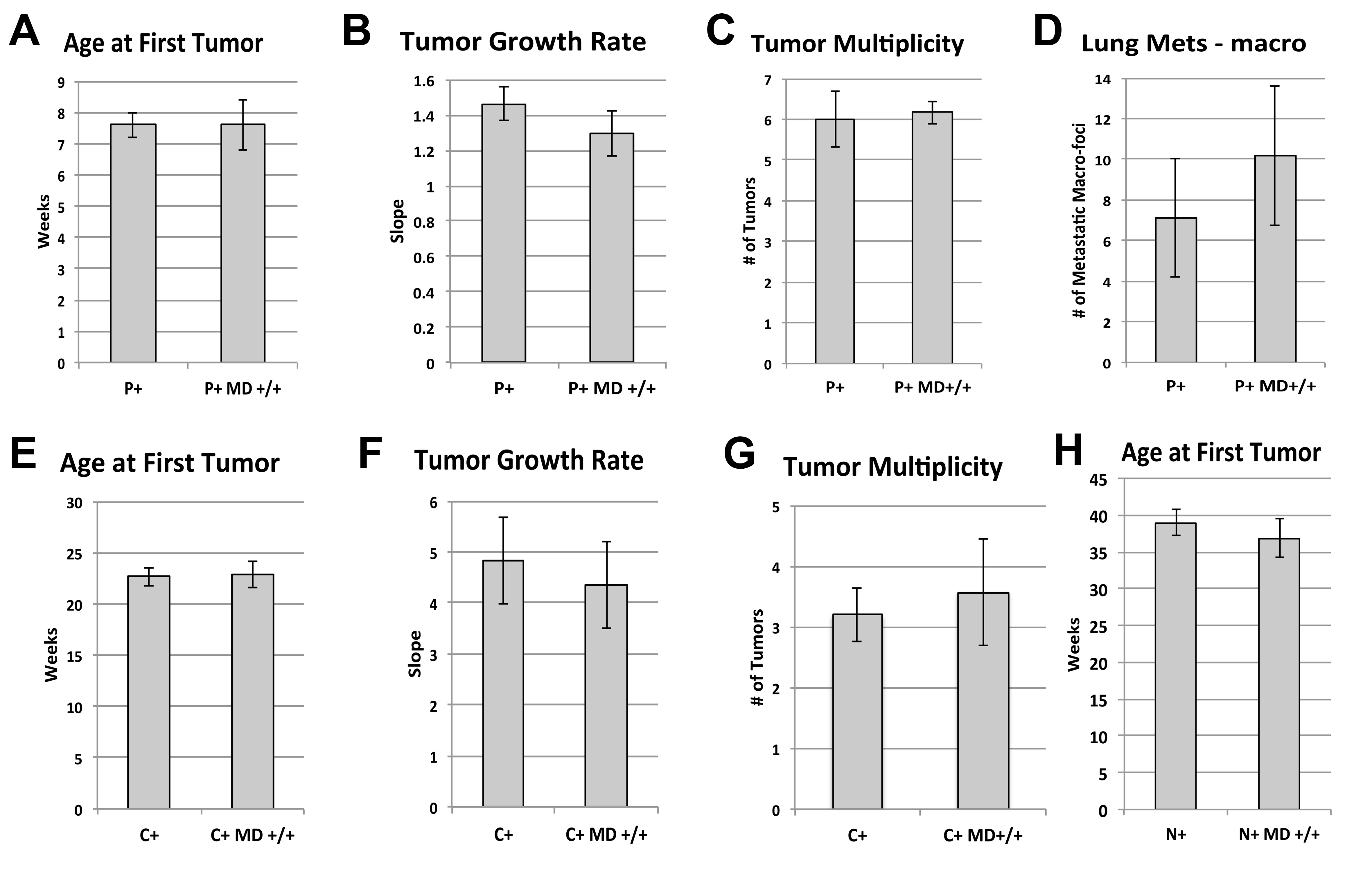
**

**Additional file 1: Figure S2:** Comparison of tumor parameters for the PyVT **(A-D)**, C3(1)-TAg **(E-G)** and neu transgenes on the FVB/N genetic background (Jackson Labs) and our MD+/+ genetic background. No differences between the genetic backgrounds were detected. Hence, we added the data for the animals with the FVB/N genetic background to the MD+/+ groups for further analysis.


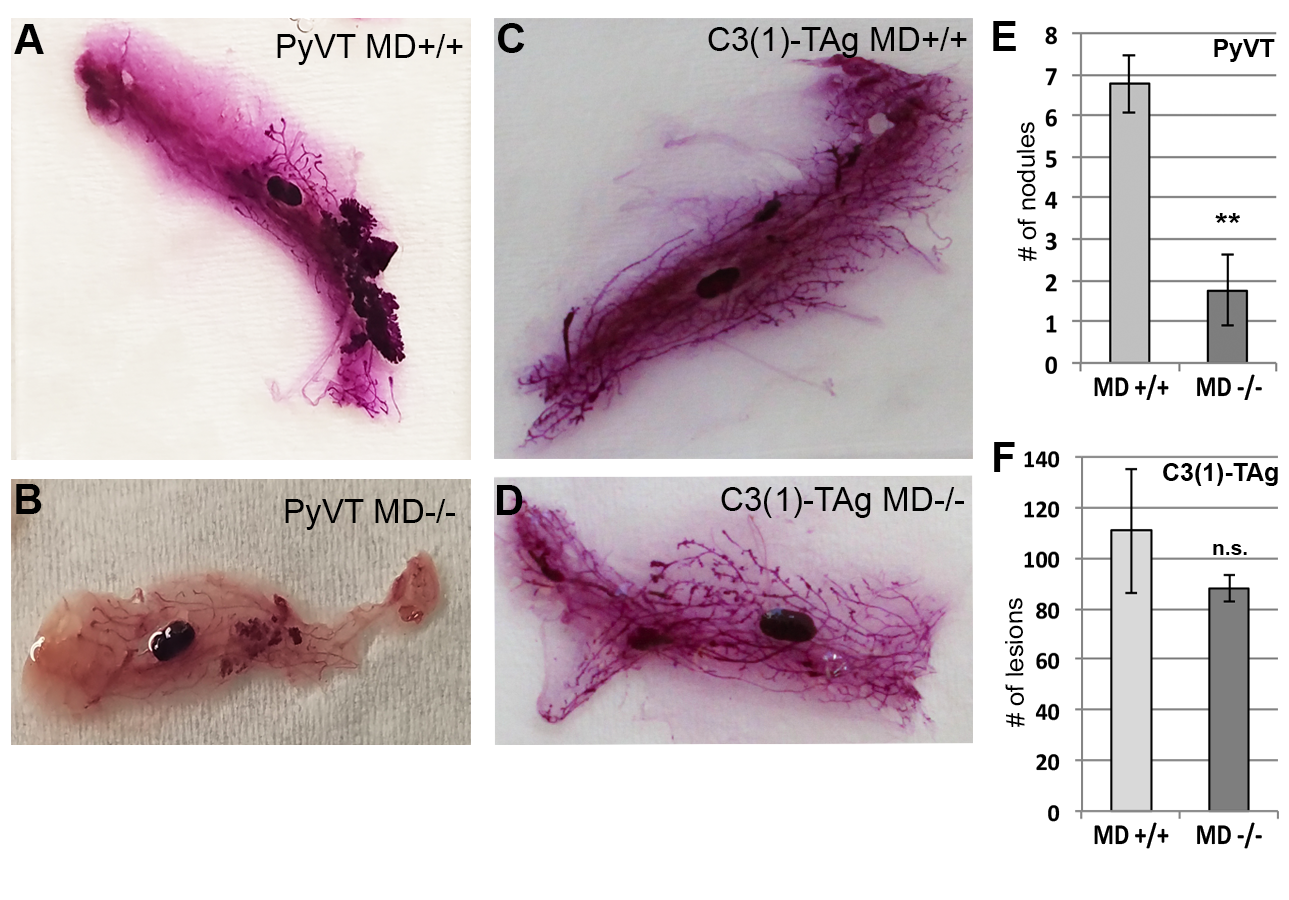


**Additional file 1: Figure S3:** Effect of MD allele on premalignant mammary glands in the PyVT and C3(1)-TAg models. **A, B)** Representative images of whole mounted mammary glands from PyVT MD+/+ (A) and PyVT MD-/- (B) mice at 46 days of age. The PyVT-induced hyperplastic expansion of the mammary epithelium is clearly visible in the MD+/+ glands, but strongly reduced in the MD-/- gland. **C, D)** Images of whole mounted mammary glands from C3(1)-TAg MD+/+ (A) and C3(1)-TAg MD-/- (B) mice at 5 months of age. Premalignant and malignant lesions are visible in both glands. **E, F)** Quantification of premalignant nodules and lesions on whole mounted mammary glands from PyVT MD+/+ (n=9) and PyVT MD-/- (n=4) mice (E), or C3(1)-TAg MD+/+ (n=3) and C3(1)-TAg MD-/- (n=4) mice (F), respectively. Graphed are averages +/- sem.

**
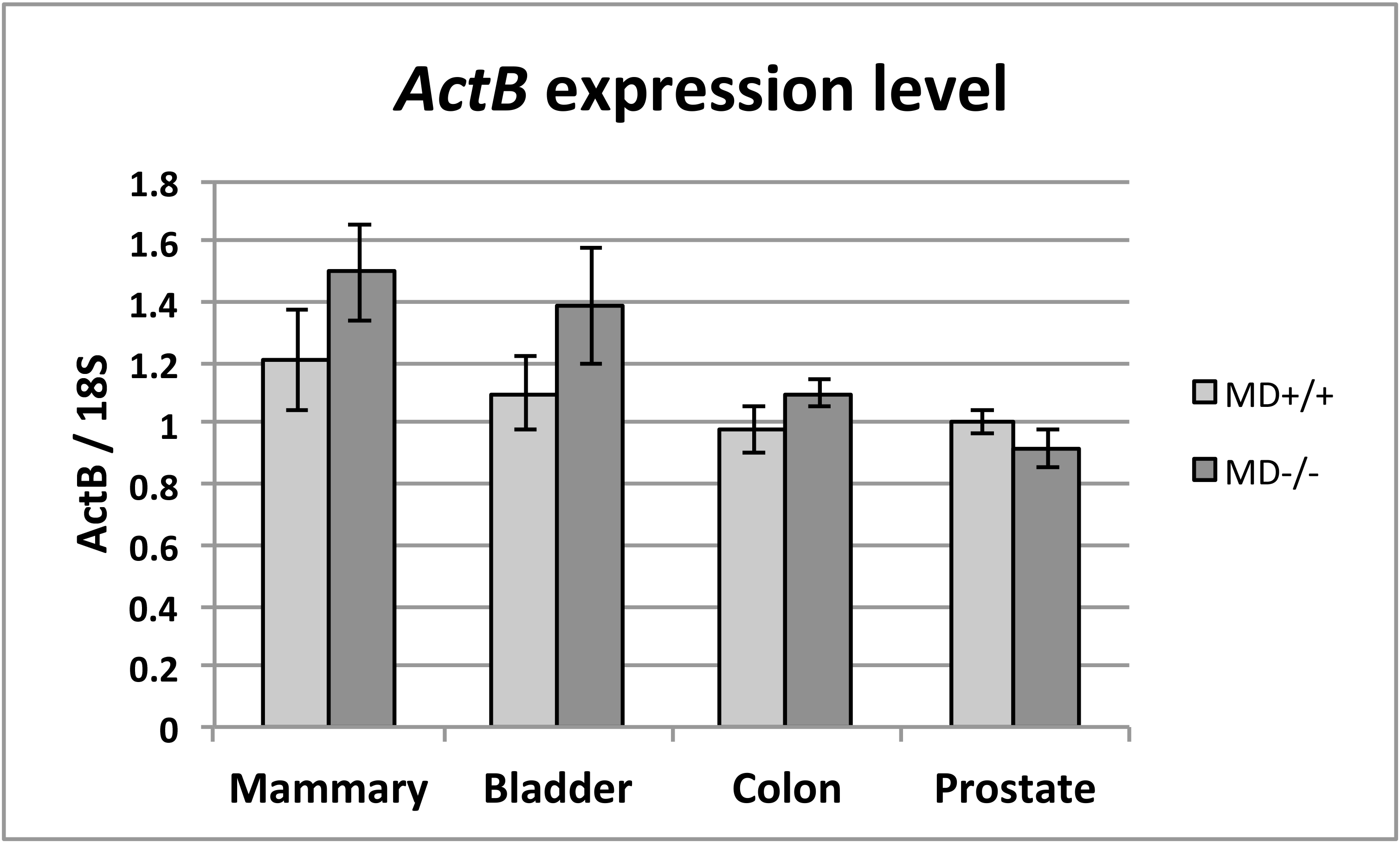
**

A

**
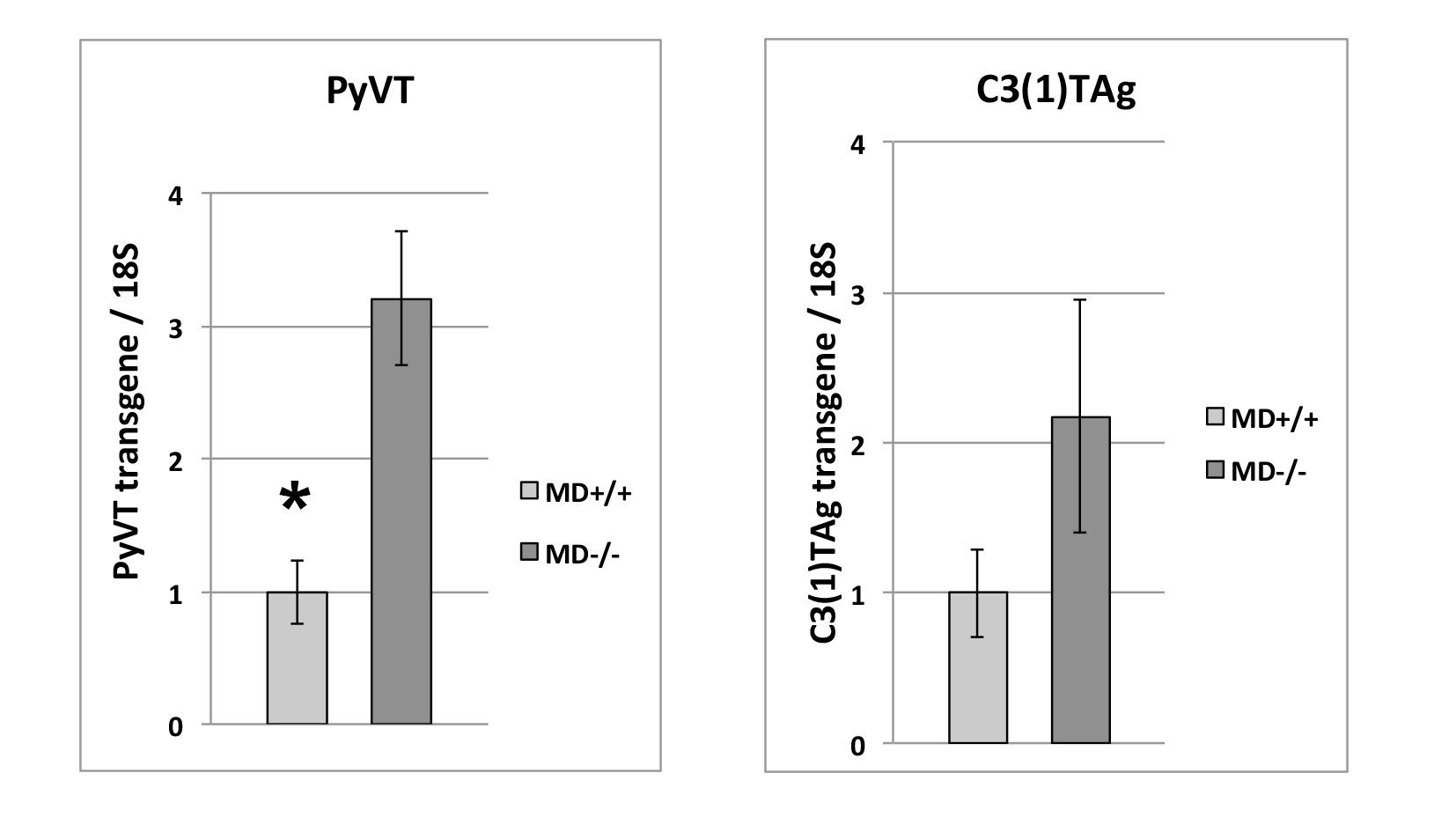
**

B

**Additional file 1: Figure S4:** Additional gene expression analysis. **A)** Comparison of housekeeping gene *ActB* transcript levels (normalized to 18S) between MD+/+ and MD-/- tissue samples. **B)** Comparison of PyVT or C3(1)-TAg transgene expression (normalized to 18S) between MD+/+ and MD-/- tumor tissue samples. Averages are shown, error bars are s.e.m. Significance (p<0.05) is indicated by an asterisk.

**
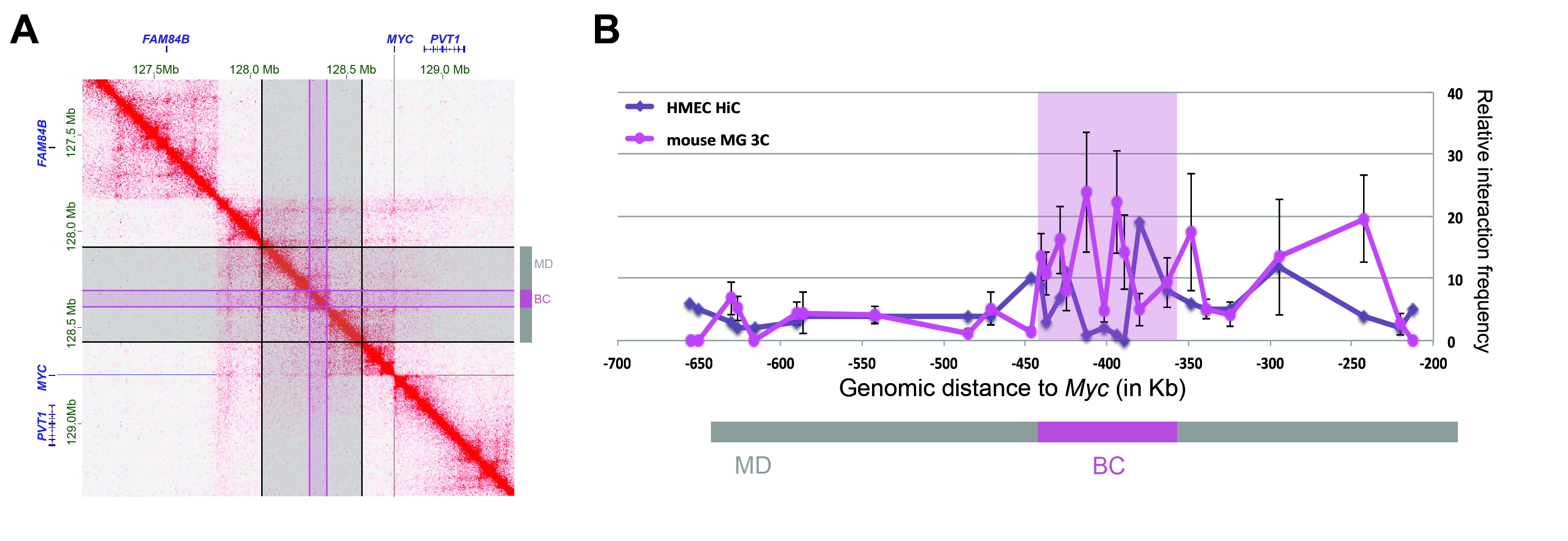
**

**Additional file 1: Figure S5: Higher-order chromatin interactions in the cancer-associated human and mouse gene desert. A)** Higher-order chromatin interaction heat map of the *8q24* locus in human mammary epithelial cell line HMEC generated using the Jukebox tool. The positions of genes *FAM84B*, *MYC* and *PVT1* are indicated. **B)** Chromatin interactions in the MD interval with the *MYC* promoter. The human interactions are shown in purple, the mouse in pink. The human interactions are derived from the vertical blue line in the heat map in panel A. The mouse interactions were determined experimentally, using the 3C assay on formaldehyde-fixed, restriction/ligation-treated MEC chromatin isolated from the mammary gland (MG) from MD+/+ mice.

**
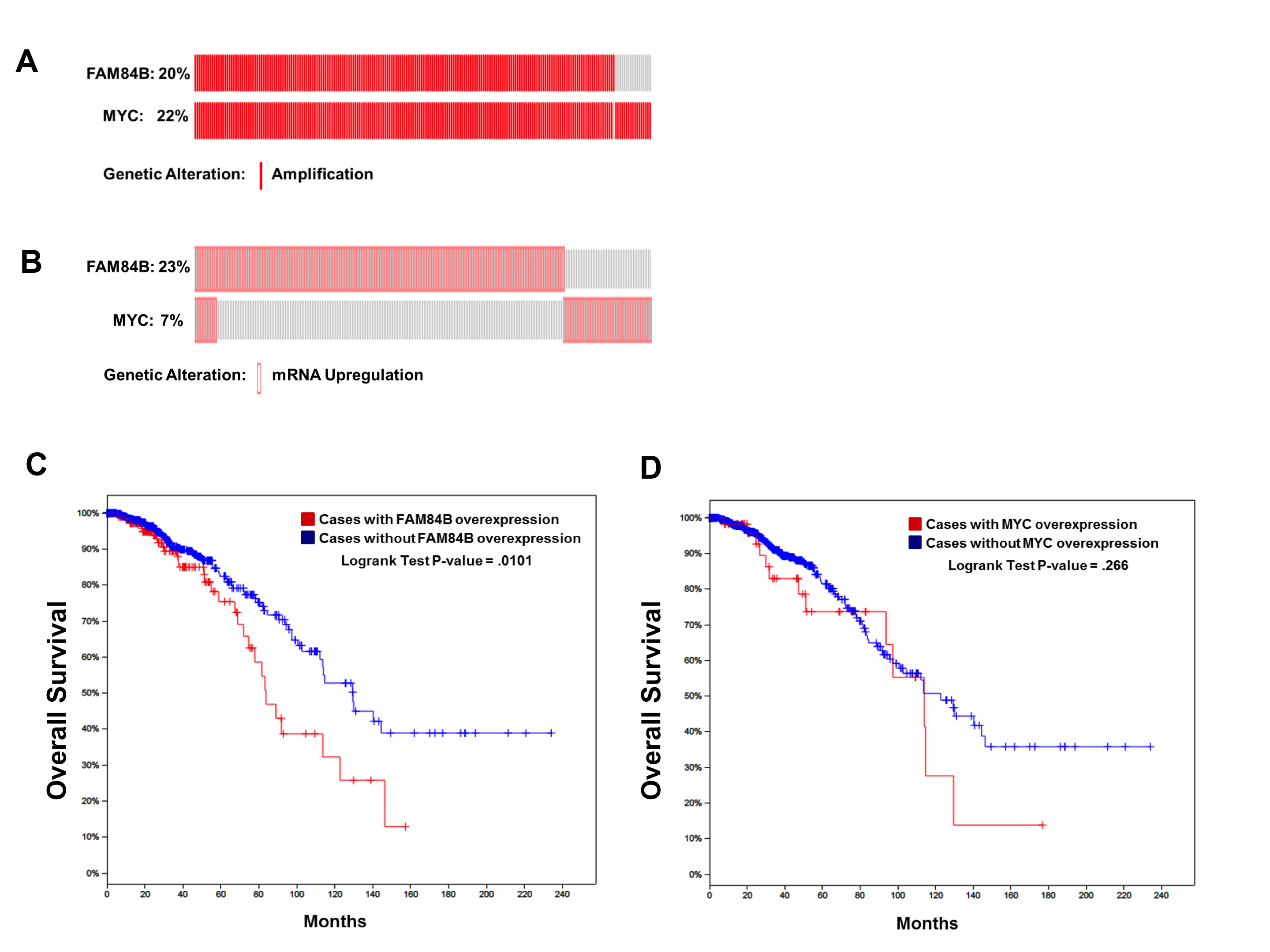
**

**Additional file 1: Figure S6: The association of *MYC* and *FAM84B* gene copy number increase and overexpression with clinical outcomes in 1105 primary breast cancers of the TCGA data set. (A)** Frequency of *MYC* and *FAM84B* copy number amplification in 35 cancer types from the TCGA bioportal database. **(B) (C)** Oncoprint showing cases from the TCGA database with *MYC* or *FAM84B* gene amplification (B) or gene expression greater than 2 standard deviations (SD) above the mean (C). **(D) (E)** Overall survival plots from the TCGA bioportal for breast cancer patients with (red plot) or without (blue plot) *FAM84B* (D) or *MYC* (E) gene overexpression (>2 SD).

B

A


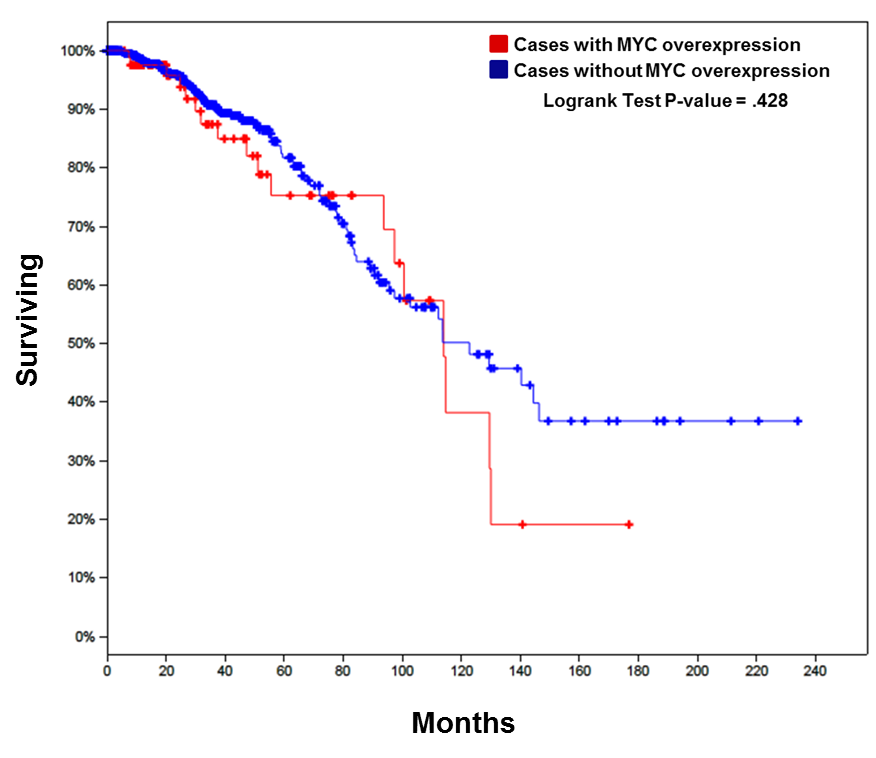

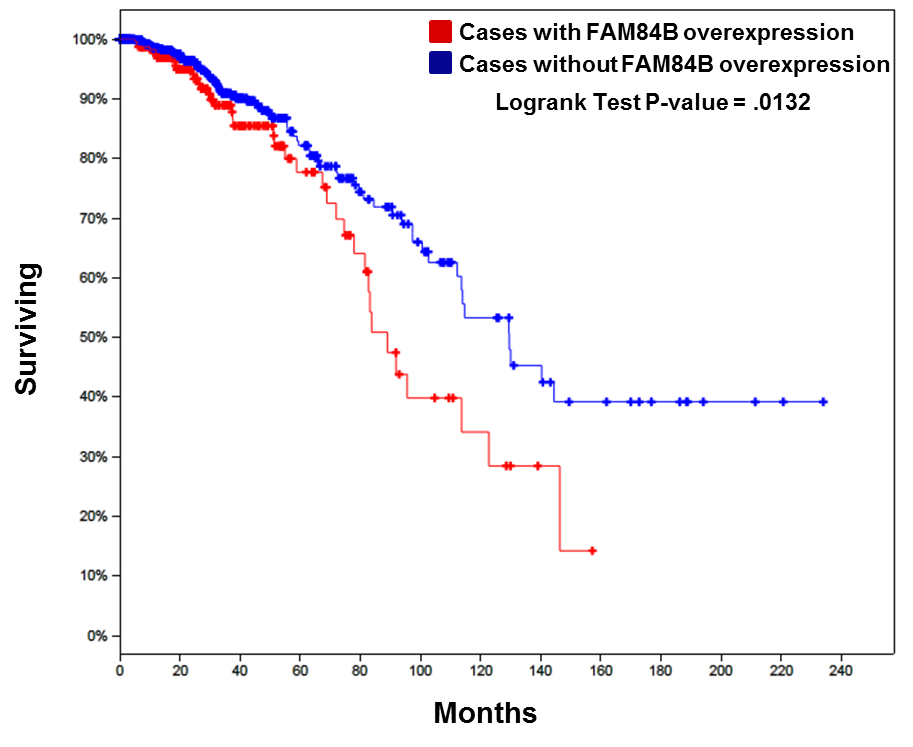


C

**
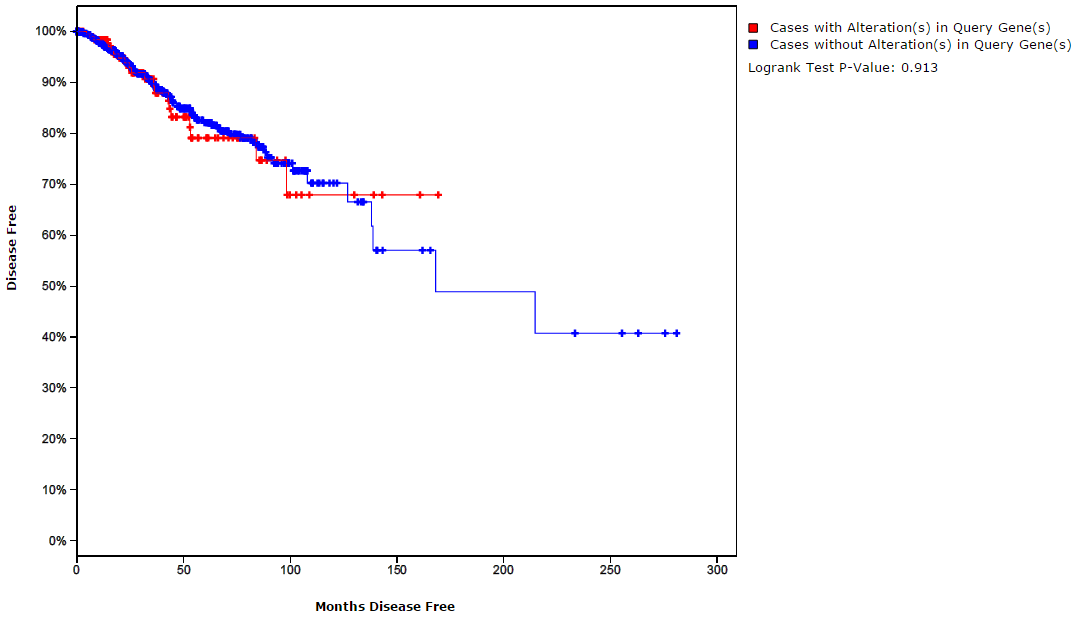

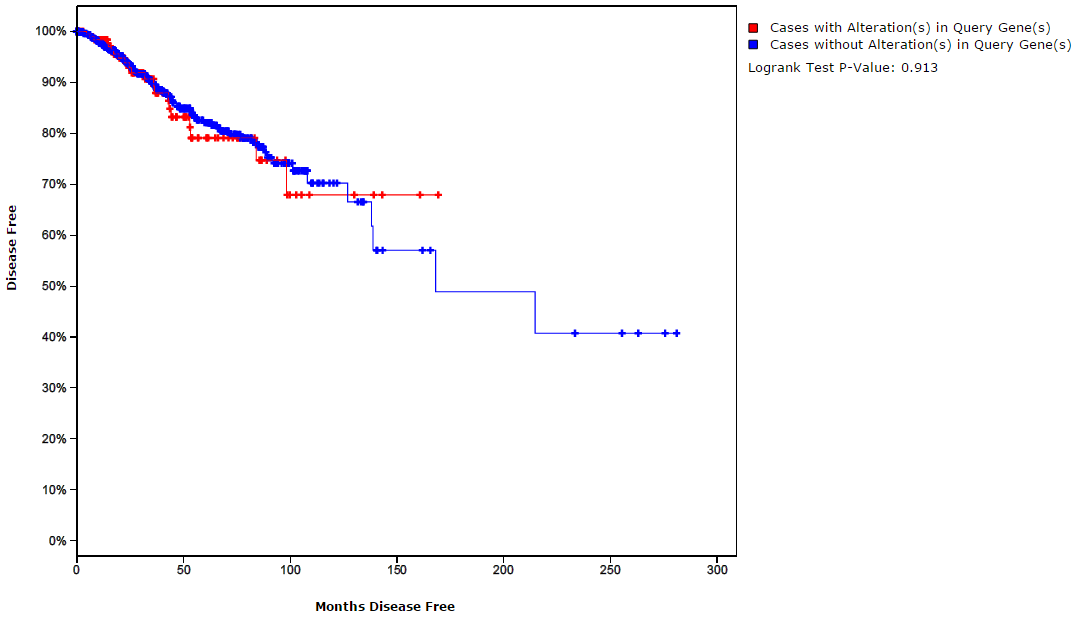
**

**Cases without FAM84B overexpression**

**Cases with FAM84B overexpression**

**Logrank Test P-value = .913**

**Months Disease-free**

**Disease-free**

**Additional file 1: Figure S7: A, B)** Overall survival plots from the TCGA cancer bioportal for breast cancer patients (n=1105), with (red plot) or without (blue plot) *FAM84B* (A) or *MYC* (B) gene overexpression at >1.5 SD. **C)** Disease-free survival plots from the TCGA bioportal for breast cancer patients with (red plot) or without (blue plot) *FAM84B* gene overexpression at >2 SD.
