# Supplementary material for: Deletion of the murine ortholog of the 8q24 gene desert has anti-cancer effects in transgenic mammary cancer models
Source: BMC Cancer. 2018 Dec 10;18:1233. doi: 10.1186/s12885-018-5109-8 (PMC6288875; doi:10.1186/s12885-018-5109-8)
Supplement: Supplementary file 2 — Table S1. List of primers, QPCR assays and BACs Table S2. 2-Way ANOVA for lung metastatic foci in PyVT mammary transplantation assay. Table S3. Total number of breast tumors in the TCGA database based on subtype. (DOCX 1631 kb) [file 12885_2018_5109_MOESM2_ESM.docx]

**Additional file 2: TABLES**

Collin Homer-Bouthiette^1,†^, Yang Zhao^1,†^, Lauren B. Shunkwiler^1^, Benjamine Van Peel^1^, Elizabeth Garrett-Mayer^2^, Rachael C. Baird^3^, Anna I. Rissman^1^, Stephen T. Guest^1^, Stephen P. Ethier^1^, Manorama C. John^4^, Patricia A. Powers^4^, Jill D. Haag^3^, Michael N. Gould^3^, Bart M. G. Smits^1,*^

^1^Department of Pathology and Laboratory Medicine, 68 President Street, Medical University of South Carolina, 29425 Charleston, SC, USA

^2^Department of Public Health Sciences, 135 Cannon Street, Medical University of South Carolina, 29425 Charleston, SC, USA

^3^McArdle Laboratory for Cancer Research, Department of Oncology, University of Wisconsin School of Medicine and Public Health, 53705 Madison, WI, USA

^4^Department of Cell and Regenerative Biology, University of Wisconsin School of Medicine and Public Health, 53705 Madison, WI, USA

*To whom correspondence should be addressed. Tel: +1 843 876 2293; E-mail: [smitsb@musc.edu](mailto:smitsb@musc.edu)

^†^ These authors contributed equally to this work

**Supplementary Table 2:** 2-Way ANOVA for lung metastatic foci in PyVT mammary transplantation assay

|  | **Lung mets - macro** | | | |
| --- | --- | --- | --- | --- |
| **WITH INTERACTION TERM** | **Df** | **Sum Sq** | **F value** | **Pr(>F)** |
| RECIPIENT | 1 | 2.813 | 0.495 | 0.486 |
| DONOR | 1 | 19.238 | 3.386 | 0.073 |
| RECIPIENT x DONOR | 1 | 6.818 | 1.200 | 0.280 |
| Residuals | 38 | 215.92 |  |  |

A donor effect was detected.

**Supplementary Table 3:** Total number of breast tumors in the TCGA database based on subtype

|  | | **>1.5 SD above the mean** | | **>2 SD above the mean** | |
| --- | --- | --- | --- | --- | --- |
| **Subtype** | **# tumor samples** | **% *FAM84B***  **over** | **% *MYC***  **over** | **% *FAM84B***  **over** | **% *MYC***  **over** |
| **All** | 971 | 28% | 10% | 23% | 7% |
| **ER+** | 594 | 33% | 7% | 27% | 4% |
| **ER-** | 174 | 16% | 21% | 13% | 14% |
| **HER2+** | 120 | **48%** | 7% | **43%** | 4% |
| **TNBC** | 82 | 11% | **29%** | 9% | **22%** |
| **Lum-A** | 201 | 31% | 2% | 24% | 0% |
| **Lum-B** | 122 | **52%** | 6% | **43%** | 3% |
| **Basal-like** | 107 | 8% | **31%** | 6% | **22%** |

ER = Estrogen Receptor, HER2 = Erbb2 receptor, TNBC = Triple Negative Breast Cancer, Lum = Luminal

The tumor subtype with highest % of *FAM84B* or *MYC* overexpressed is printed in bold.
